# A Comparative Study of Conductive 3D Printing Filaments for Electrochemical Sensing Applications Pretreated by Alumina Polishing, Electrochemical Activation, and Electrodeposition of Au Nanoparticles

**DOI:** 10.1021/acselectrochem.5c00240

**Published:** 2025-10-28

**Authors:** Shakir Ahmed, Enock G. Arthur, Tanner Obrzut, Ricoveer Shergill, Alexa Williams, Kelvin Wamalwa, Zackary D. Epright, Cameron Darvish, Yousef Khatib, Wanlu Li, Bhavik A. Patel, Glen D. O’Neil

**Affiliations:** a Department of Chemistry and Biochemistry, 8087 Montclair State University, Montclair, New Jersey 07043, United States; b School of Applied Sciences, 1947 University of Brighton, Brighton BN2 4GJ, U.K.; c Centre for Lifelong Health, 1947 University of Brighton, Brighton BN2 4GJ, U.K.; d Sokol Institute for Pharmaceutical Life Sciences, 8087 Montclair State University, Montclair, New Jersey 07043, United States

**Keywords:** Composite electrodes, 3D-printed electrode, partially blocked electrode, surface activation, structure−property relationships

## Abstract

3D-printed electrochemical devices have gained tremendous attention recently because they are highly customizable platforms for analysis and energy storage that can be produced using simple, inexpensive components in a wide variety of settings. 3D-printed electrochemical sensors, fabricated from carbon-loaded conductive thermoplastics, enable decentralized production of electrochemical devices that, if optimized, could be widely distributed. Achieving this goal requires a comprehensive understanding of the electrochemical behavior of these filaments. Here, we investigated how the electrochemical behavior of three commercial filaments was affected by alumina polishing, electrochemical activation in 0.5 M NaOH, and electrodepositing Au nanoparticles (NPs). The goal of this study is to characterize if/how these commonly used pretreatments affect different filaments. The study is not an exhaustive combination of all filaments and pretreatment options. We characterized the physical properties of each filament/pretreatment using thermogravimetric analysis, scanning electron microscopy, and Raman microscopy measurements. We then benchmarked the background electrochemical processes (capacitance and solvent window), the peak current response versus scan rate, and the peak potential separation of two common outer-sphere redox species (ruthenium hexamine and ferrocene methanol) for each filament under each pretreatment (*i.e.*, nine total conditions). We subsequently investigated how the filaments responded to inner-sphere redox couples that were surface sensitive (ferrocyanide oxidation), dependent on surface adsorption (dopamine oxidation), and sensitive to surface oxides (Fe^2+^ oxidation). The data collectively underline the complexity of electrodes fabricated from conductive 3D printing filaments and highlight several important considerations that should be addressed when interpreting the electrochemistry of such materials. First, we present evidence that these materials behave as partially blocked electrodes, which complicates interpretations of electrochemical data. We also found that the outer-sphere electrochemical reactivity on a given filament was largely consistent regardless of pretreatment. The important variable for assessing outer-sphere electron transfer was the uncompensated resistance (*R*_u_), which varies depending on the filament material, electrode size, and contact method. Finally, we observed that the selected filaments do not respond to pretreatments identically when tested against inner-sphere redox species, suggesting that a variety of treatments should be evaluated when assessing conductive 3D-printed filament electrodes.

## Graphical abstract

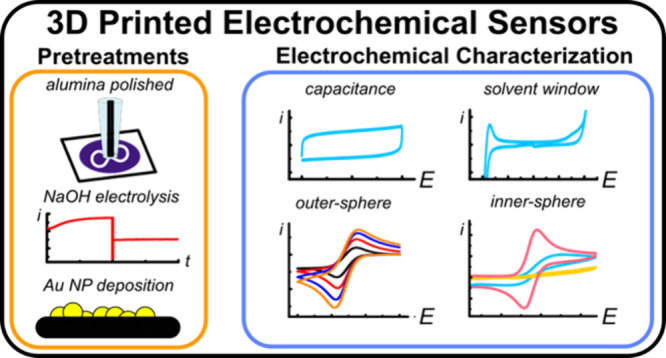

## Introduction

3D printing has emerged as a promising technique to produce electrochemical sensors and devices of nearly any geometry.^1–4^ The motivation for employing 3D printing for electrochemical applications is that customized devices are easily produced at low cost using inexpensive benchtop equipment,^5–7^ opening the potential for decentralized fabrication at the point of use.^8^ 3D printing is also highly advantageous for iterative design of new technologies, individual production of highly customized components, or mass production of inexpensive sensors and devices. Because of this versatility and the high performance of 3D-printed electrodes, there are many excellent examples (and reviews^1–4,9–16^ ) of 3D-printed electrochemical sensors for biological,^17–24^ environmental,^25–30^ forensic,^31–33^ pharmaceutical,^34,35^ and energy storage applications.^36–45^

While numerous materials and 3D printing techniques are available for producing electrochemical sensors, fused deposition modeling (FDM) printing of conductive thermoplastics is the most widely used due to the low upfront costs of desktop 3D printers (≈500–3000 USD)^3,12^ and high performance of the thermoplastic electrodes. The most promising 3D printable electrochemical sensing filaments are composites of an extrudable thermoplastic—typically poly­(lactic acid) (PLA) or acrylonitrile butadiene styrene (ABS)—mixed with conductive carbon micro- or nanoparticles,^46^ and share many of the same properties of classical composite electrodes (*e.g.*, carbon paste electrodes). While numerous examples of custom-synthesized filaments designed for specific applications have appeared in the literature,^47–49^ the majority of 3D-printed electrochemical sensors are fabricated using commercial filaments.^9^ Several variables influence the electrochemical performance of composites,^48,50^ including the allotrope/quality of carbon,^51,52^ the type of insulating binder,^52–54^ the presence of plasticizer in the filament,^55^ the mass loading of the conductive material,^53^ the uniform distribution of conductive particles within the composite, the surface treatment before use,^56,57^ the age of the filament,^58^ and the degree of water ingress into the filament.^59^ Custom-synthesized filaments can have high performance compared with commercial filaments, even without pretreatment. However, these materials are less accessible given they are made in-house.

Compared with custom-synthesized filaments, the formulations of commercial conductive filaments are fixed (*i.e.*, type of binder, type and mass loading of carbon, etc.). Therefore, the surface treatment of the electrodes have a significant impact on the performance of the electrodes and is the primary method for tuning their performance (note that all 3D-printed electrodes can benefit from surface treatments). This is because the electrochemistry of 3D-printed electrodes is influenced by the reactivity of the surface (determined by the number of exposed particles and their electrochemical activities), the mass transfer of reactant/product to/from the surface, the resistivity of the composite, and the contact resistance of between the electrode and the potentiostat.^60^ It is important to note that it is impossible to distinguish between slow kinetics and high uncompensated resistance in a single voltammetric experiment. Recent work has shown that the “as-printed” interfaces typically have poor electrochemical performance, characterized by wide peak-to-peak separations (Δ*E*p) towards outer- and inner-sphere redox couples, which is consistent with slow heterogeneous electron transfer (HET) and/or high uncompensated resistance.^61^ While careful control of the contact resistance and electrode geometry can mitigate (to varying degrees) the uncompensated resistance, the electrode surface condition of as-printed interfaces requires attention. The most widely cited hypothesis explaining the poor surface condition is that during extrusion the insulating thermoplastic coats the embedded conductor material rendering it electrochemically inactive (see, for example, refs. 31,32,62 ). As a result, the majority 3D-printed sensors require an pretreatment step to improve the electrode behavior. Many approaches to improve the surfaces are available; most pretreatments focus on removing the thermoplastic to expose more of the underlying conductive particles and activating the particles towards a specific reaction of interest.^20,32,56,57^ Finally, more active catalyst films or particles can be deposited on the surface of the sensor to increase the electrochemical activity.^8,22,25^ Recently, the Patel group has introduced methods for “pre-activating” the surfaces by soaking the filaments in NaOH prior to printing^63^ and electrodepositing Prussian blue nanoparticles on the filaments prior to extrusion.^64^ A significant number of papers have explored how the material properties of 3D-printed electrodes influence the electrochemical properties, including in-depth studies of how the nature of the electrochemical and/or solvent pretreatment(s),^32,56,57,65,66^ the 3D printing orientation,^67^ surface patterning,^68^ print parameters,^69^ handling of the filament before printing,^63^ filament age,^58^ carbon allotrope type,^70^ and commercial source of filament influence the behavior of 3D-printed electrodes. However, the majority of these studies use a single type of filament to study the effect of various pre-treatments or use one pretreatment with different commercial filaments; a study that systematically compares how different filaments are impacted by different pretreatments is missing from the current literature.

In this study, we investigated how the electrochemical behavior of three commercial filaments was affected by alumina polishing, electrochemical activation in 0.5 M NaOH, and electrodepositing Au nanoparticles (NPs). Our primary motivation was to characterize and understand if/how these commonly used pretreatments affect different filaments. We focused our study on three commercially available conductive PLA filaments (Amolen, BlackMagic 3D, and ProtoPasta) due to widespread use in the literature. We chose three representative pretreatments (alumina polishing, electrochemical activation in 0.5 M NaOH, and deposition of Au nanoparticles) that have been shown to produce high-quality interfaces for electrochemical sensing. Note that the electrodes were first polished with alumina prior to both Au NP deposition and electrolysis in NaOH. While each of these filaments have been studied independently, the present work provides a systematic comparison each filament and pre-treatment. By subjecting each filament to identical pretreatments and electrochemical characterizations, we reveal key differences in behavior that are not apparent from isolated studies. We characterized the physical properties of each filament/pretreatment using scanning electron microscopy (SEM), thermogravimetric analysis (TGA), and Raman microscopy measurements. We then benchmarked the background electrochemical processes (capacitance and solvent window), peak current behavior, and the peak potential separation (Δ*E*p) of two common outer-sphere redox species (ruthenium hexamine and ferrocene methanol) for each filament under each pretreatment (*i.e.*, nine total conditions). We subsequently investigated how the filaments responded to inner-sphere redox couples that were surface sensitive (ferri-/ferrocyanide), dependent on surface adsorption (dopamine), and sensitive to surface oxides (Fe^2+/3+^).

## Experimental Section

### Materials and Solutions

Hexaammineruthenium­(III) chloride (98%) was purchased from Acros Organics and used without purification. All other reagents were purchased from Fisher Scientific, were of ACS reagent grade or better, and were used as received. All solutions were prepared using deionized water with a resistivity of 18.2 MΩ·cm at 25° C (Millipore Simplicity).

Commercialized 3D printable carbon composites were obtained from ProtoPasta (USA), BlackMagic 3D (USA), and Amolen (China). We note that at the time of submission, BlackMagic 3D is no longer commercially available. Amolen and ProtoPasta both use carbon black as the carbon source while Blackmagic 3D uses graphene as the major carbon source. The binder for all three filaments was PLA. Some selected properties of each filament are presented in Table 1.

**Table 1:** Selected Properties of Amolen, BlackMagic 3D, and ProtoPasta Filaments

Filament	Carbon	% wt carbon^a^	Binder	Quoted Resistivity, Ω·cm
BlackMagic 3D	Graphene	14 ± 2	PLA	0.6 ^71^
ProtoPasta	Carbon Black	24 ± 2		30 (in plane); 115 (through plane)^72^
Amolen	Carbon Black	23 ± 2		1.5 ^73^

a Measured using TGA.

### Electrode Preparation

In this study, we aimed to compare extruded filaments while excluding variables caused by the printing process including surface roughness, porosity, or voids within the printed structure.^67,74^ To achieve this goal, we prepared disk electrodes of extruded filament in a form factor that is ubiquitous in electrochemical studies (Figure S1a).^16^ This configuration has several advantages including low uncompensated resistance, utilization of waste filament from priming the printer, elimination of orientation effects on the printed part, and a form factor that enables reproducible refreshing of the electrode surface via polishing with alumina slurries. Briefly, 100 mm of filament was extruded through a 0.5 mm stainless steel nozzle at 215 °C into open air. The filament was cut into 2.5 cm pieces before an Ohmic contact was made using Ag paste and a Cu wire. The contacted filament assembly was potted in a 100 μL Eppendorf pipette tip previously filled with 2-part Epoxy (JB Weld) while pulling the filament forward and backwards through the epoxy to ensure the filament was completed coated. The end was cleaved with a clean scalpel blade approximately 1 cm from the location of the filament/Cu wire contact to expose a disk electrode surface. Therefore, the conduction pathway between the electrode surface and ohmic connection was ∼1 cm. After cleaving the excess filament, all electrodes were subsequently wet sanded with 600- and 1200-grit carbide papers, rinsed and polished with 1.0, 0.3, and 0.05 μm slurries (CH Instruments), taking care to rinse the electrodes carefully between each slurry. After the final polishing step, residual alumina particles were removed by polishing on a wet polishing pad without alumina. After the initial preparation and polishing, the electrode surfaces were refreshed before each experiment by polishing with 0.05 μm alumina slurries. Three conditions were investigated in this study: polishing with alumina slurries, electrodeposition of Au nanoparticles (NPs), and electrolysis in 0.5 M NaOH. Note that the electrodes were first polished with alumina prior to both Au NP deposition and electrolysis in NaOH. For Au NP deposition, we initially screened several Au NP deposition protocols from the literature but found they gave unsatisfactory responses and were unstable. Au NPs were electrodeposited from an electrolyte containing 2 mM HAuCl_4_ and 0.1 M HCl using a two-step voltage pulse. The first step is applied briefly (2 seconds) with a large overpotential (*E*_dep_ = −1.0 V vs. SCE) to nucleate Au NPs on the surface. The second step (18 seconds) uses a lower overpotential (*E*_dep_ = −0.4 V vs. SCE) to grow the seed particles. After electrodeposition, we cycled the Au NP deposited electrodes in 0.1 M H_2_SO_4_ from 0 V to +1.6 V to −0.3 V for 20 cycles (each cycle ended at 0 V). For activation of the filaments in NaOH, we followed the widely-used protocol from Richter and co-workers.^20^ Briefly, the electrodes were cut and polished as described above before being immersed in a 0.5 M NaOH solution. The electrodes were polarized at +1.4 V for 200 seconds before the polarity was switched to −1.4 V for 200 seconds. The electrodes were thoroughly rinsed with DI water before they were used for further experiments. Electrodes were re-used after a pretreatment by repeating the entire polishing procedure, starting with wet-sanding, as described above.

### Electrochemical Measurements

All electrochemical experiments were performed in a three-electrode cell with a Ag/AgCl­(saturated KCl) or saturated calomel (SCE) reference electrode and Pt wire counter electrode. All potentials in the main text are referenced to a saturated Ag/AgCl by measuring the potential difference between the two reference electrodes (ca. 44±2 mV). Unless otherwise stated, the first CV was used for presentation and analysis of the data. The potential was controlled using a CH660C or CH760E potentiostat (CH Instruments, Austin, TX, US) interfaced through a PC.

Capacitance measurements were performed in 0.1 M KNO_3_ using cyclic voltammetry (CV) over the scan range from ±0.1 V at 0.1 V s^–1^. Scans began at -0.1 V, with the first scan in the positive direction. Capacitance was calculated using eq 1:

$$C_{\mathrm{dl}}=\frac{\Delta i}{2vA}$$ 1

where *C*_dl_ is the double layer capacitance (in F cm^–2^), Δ*i* is the difference in current between the forward and reverse scans (measured at 0 V from the third cycle), *v* is the scan rate (V s^–1^), and *A* is the geometric area of the electrode (cm^2^). Note the third CV cycle was used because the CVs stabilized by the third cycle.

Solvent window measurements were carried out in 0.1 M KNO_3_ using CV by scanning over the approximate potential range from ±2.3 V at 0.1 V s^–1^. Scans began at 0.0 V with the first scan in the negative direction. The solvent window was determined by measuring the difference in anodic and cathodic potentials where the current exceeded 0.4 mA cm^–2^ on the forward sweep.^75^

Measurements of outer-sphere (Ru­(NH_3_)_6_^3+^, FcMeOH) and inner-sphere species (Fe­(CN)_6_^4–^, Fe^2+^, dopamine) were performed using CV. FcMeOH, Ru­(NH_3_)_6_^3+^, and Fe­(CN)_6_^4–^ were measured in an electrolyte also containing 0.1 M KNO_3_; Fe^2+^ was measured in an electrolyte also containing 0.1 M HClO_4_; dopamine was measured in 1X PBS, which has the following composition: 137 mM NaCl, 2.7 mM KCl, 10 mM Na_2_HPO_4_, and 1.8 mM KH_2_PO_4_. For these experiments, solutions were not purged of dissolved oxygen prior to data collection. This choice allowed us to directly observe whether the filaments exhibited catalytic activity toward oxygen reduction. When significant oxygen reduction currents were observed, supplementary experiments with Ar purging were performed to confirm the impact of dissolved oxygen. For outer-sphere couples, measurements were carried out at four scan rates (0.1, 0.25, 0.5, and 0.75 V s^–1^) to determine if the current response was diffusion-limited. CV measurements were performed at 0.1 V s^–1^ for inner-sphere couples.

### Thermal Gravimetric Analysis (TGA)

The thermal analysis was done with a thermogravimetric analyzer (TA Instruments, SDS 650) under nitrogen atmosphere over a temperature range of 30–800 °C with a heating rate 10 °C/min. The sample weight was about 10 mg. Each filament was analyzed in triplicate and the results are reported as the mean ± one standard deviation.

### Microscopy Measurements

SEM was performed using a Hitachi S-3400N SEM in secondary electron mode using a 25 kV accelerator voltage. To prepare samples for SEM imaging, 1–2 mm was cleaved from the electrode surface after the pretreatment was carried out. The electrode tips were mounted on carbon tape on an SEM stub and loaded into the vacuum chamber for imaging. Raman spectra were collected on a Renishaw InVia microscope with a 633 nm HeNe laser that was focused on the sample through a 20× objective. The Raman microscope was calibrated with a Si wafer before measurements. The laser power was set to 5% (≈2 mW) and spectra were acquired with 180 s exposure time. Backgrounds were removed in OriginLab using the Peak Analysis tool, cosmic rays were removed using the cosmic ray removal algorithm included with the Renishaw software, and peaks were normalized versus the G band peak after background subtraction. AFM was performed using a Bruker Dimension Icon AFM. The images were collected in ScanAsyst mode using a SCANASYST-AIR probe (7 nm radius, k = 0.4 N/m, f = 70 kHz). The imaging rate was between 0.1 and 0.5 Hz with a resolution of 512 samples/line. All images were 25 μm × 25 μm.

### Statistical Analysis and Data Representation

TGA analysis was performed in triplicate for each material. SEM data is presented as one image (of three) collected per sample. Nanoparticle size and particle density estimates are collected from the SEM images using ImageJ. In these analyses, the particles are assumed to be spherical and we omitted particle aggregates from size estimates and counting. The presented cyclic voltammograms are representative of data from (at least) three separately prepared electrodes. The quantitative parameters extracted from TGA, SEM, and CV data are reported as the mean ± one standard deviation (unless otherwise noted). Statistical comparisons were made using confidence intervals, two-tailed Students t-tests or one way ANOVA with *post hoc* Bonferroni corrections, depending on the circumstances. The bar graphs represent the mean value and error bars represent one standard deviation (unless otherwise noted). Significance testing is represented using asterisks on each graph where ***: *p* < 0.001, **: *p* < 0.01, and *: *p* < 0.05. The absence of asterisks means no significance was observed (*p* > 0.05).

## Results and Discussion

### Description of Conductive Filaments, Preparation of Electrodes, and Study Design

We obtained three conductive filaments from Amolen, BlackMagic 3D, and ProtoPasta. Each of these filaments is a composite composed of PLA binder and conductive carbon particles. Amolen and ProtoPasta contain carbon black, while BlackMagic 3D primarily uses graphene. We note that at the time of submission, BlackMagic 3D is no longer commercially available; however, an alternative graphene/PLA filament is available from Colfeed, and appears to have similar electrochemical characteristics to BlackMagic 3D.^70^ Given the fact that BlackMagic 3D is no longer available, the results obtained herein are used as a comparative benchmark for graphene-based conductive filaments more generally. We chose to constrain our investigation to PLA-based composites over those based on nylon, ABS, or other polymers because PLA-based filaments are the most widely used. A summary of the selected physical properties of each filament is presented in Table 1.

To effectively compare the electrochemical behavior of filaments, it was important to consider the form factor of the fabricated electrodes. Here, we prepared simple disk electrodes by sealing extruded filament strands in insulating two-part araldite epoxy within a 100 μL pipette tip, similar to previous work.^17^ Electrodes were prepared at both Montclair and Brighton to evaluate the lab-to-lab reproducibility of the method. This configuration has several advantages including high reproducibility, ease of preparation, and low cost. An additional advantage is that we can eliminate complicating variables associated with print orientation, printing speed, and printing temperature which are commonly observed with 3D-printed electrochemical sensors.^67^ To ensure that the exposed area of the 3D-printed filament was co-planar with the surrounding insulator and there were no voids at the interface of the filament and epoxy, we imaged each electrode after fabrication (Figure S1). We observed a tight seal between the epoxy insulation and conductive filament for all electrodes used herein, indicating that the electrode geometry was a co-planar disk. We measured the diameter of each electrode using ImageJ and determined the geometric area (*A* = π­(*d*/2)^2^; Table S1). The areas of the BlackMagic 3D and ProtoPasta electrodes were similar (2.00(±0.02) × 10^–3^ and 1.82(±0.08) × 10^–3^ cm^2^, respectively), while the Amolen electrode was significantly larger (3.0(±0.2) × 10^–3^ cm^2^). We attribute the differences in area to changes in the extruded diameter of each filament, which is impacted by the material properties of the composite.

The goal of this study is to investigate how the electrochemical behavior of three commercial filaments was affected by alumina polishing,^77^ and subsequently electrochemical activation in 0.5 M NaOH,^20^ and electrodepositing Au nanoparticles^8^ (NPs). We performed the latter two pretreatments on a polished electrode surface to ensure that the geometry of the electrode was well-defined and characterized. As such, the polished condition serves as our “baseline” measurement and point of comparison for the NaOH electrolysis and Au NP deposition pretreatments. We selected three filaments that have been widely used in the electrochemical sensing literature: BlackMagic 3D, ProtoPasta, and Amolen. While there are a large number of pretreatments available,^76^ we chose three representative protocols that are widely available and familiar to the electrochemical sensing community.

### Physical Characterization of Printed Materials

We first evaluated the physical properties that most significantly impact the electrochemical response, namely the thermal stability, morphology of the electrode surfaces, and the disorder of the carbon allotrope.

#### Thermogravimetric Analysis

We performed TGA to determine the carbon content of each filament and to understand the nature of the interactions between the carbon particles and PLA in the composite. Figure 1a shows representative TGA curves for Amolen, BlackMagic 3D, and ProtoPasta filaments. The filaments each show a significant weight loss at temperatures between 320-500 °C, consistent with the decomposition of the PLA filament. We first assessed the residual carbon content in each filament using the weight remaining at temperatures >500°C, and the values are summarized in Table 1. The Amolen, ProtoPasta, and BlackMagic 3D filaments were 23±2%, 24±2%, and 14±2% carbon, respectively. These are consistent with previously reported values for each filament.^49,58,78^

**Figure 1:**
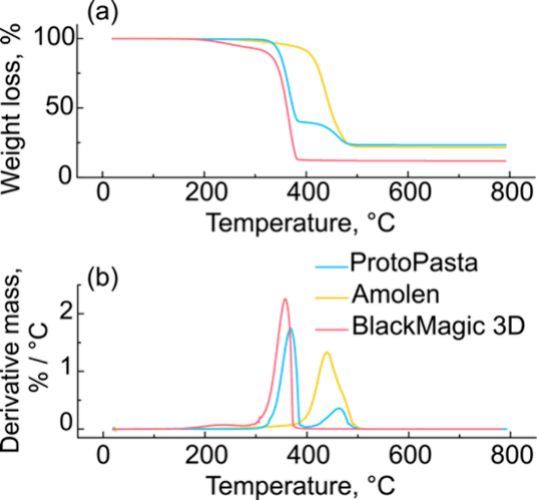
(a) Thermogravimetric analysis and (b) differential gravimetric analysis of Amolen, ProtoPasta, and BlackMagic filaments under a nitrogen atmosphere. Samples were ∼10 mg and the temperature ramp rate was 10 °C min^–1^.

TGA was also used to understand the interaction between the carbon and polymer within composite materials. In the transition temperature range of the TGA curves (∼200–500 °C), each filament shows multiple mass losses, as shown in the derivative mass curves in Figure 1b. The BlackMagic samples show a small weight loss (∼5%) at around 230 °C and a larger weight loss (∼80%) at 360 °C. We attribute these two peaks to the degradation of oxidized functional groups on the graphene and the degradation of PLA, respectively.^58,79^ The ProtoPasta filament shows a large weight loss at ∼370 °C, attributed to PLA degradation, and a smaller loss at ∼465 °C, attributed to an additive/plasticizer in the ProtoPasta filament. The Amolen filament shows a significant weight loss at 440 °C, with a small shoulder at ∼480 °C, attributed to PLA and a crosslinking agent. We attempted to dissolve the PLA using tetrahydrofuran (THF; Figure S2), but observed that Amolen was resistant to dissolution after 90 min of sonication and even after several months in the solvent. These data show that the thermal stability of the three filaments differs dramatically, likely originating from differing additive compositions.

#### SEM Characterization of Electrode Morphology

A qualitative analysis of each electrode’s surface morphology was carried out using SEM. Figure 2 shows SEM images of Amolen, BlackMagic 3D, and ProtoPasta filaments prepared with three representative pretreatments: polishing with alumina, electrolysis in 0.5 M NaOH, and electrodeposition of Au NPs. First, consider Figure 2i (top row), which shows the polished electrode surfaces which serve as our “baseline” treatment. Polishing is the most common electrode pretreatment and is used throughout the electrochemical literature to refresh electrode surfaces and, therefore, serves as an important control preparation in this study.^77^ The surfaces of all three electrodes are qualitatively similar, showing very little macroscopic roughness but demonstrating surface imperfections caused by imperfect wearing of the composite during polishing.

**Figure 2:**
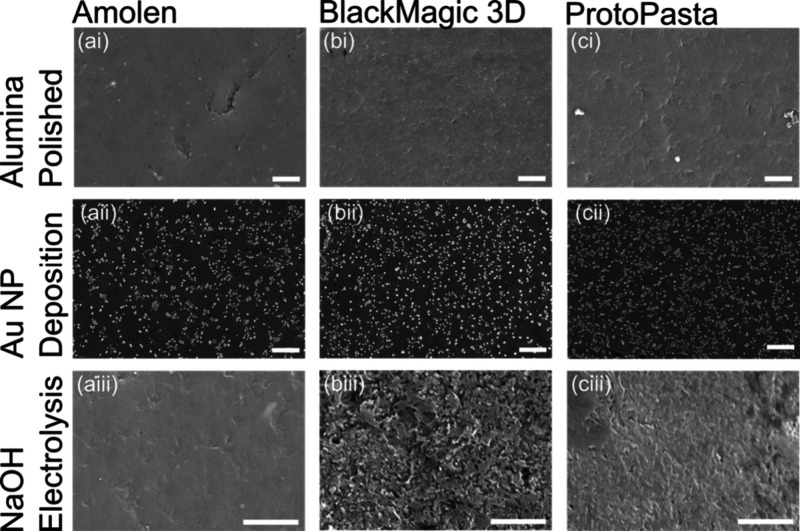
SEM characterization of 3D-printed filament electrodes prepared using (a) Amolen, (b) BlackMagic 3D, and (c) ProtoPasta filaments. Each filament was treated by (i) polishing with 0.05 μm alumina slurry, (ii) modification with Au NPs, and (iii) polishing and subsequent electrochemical treatment in NaOH. Scale bar = 5 μm.

The SEMs in Figure 2ii (middle row) demonstrate that the surfaces can be modified with Au NPs and that the size, density, and distribution of the NPs are qualitatively similar. We estimated the density and diameter of the nanoparticles using the particle sizing algorithm in ImageJ software (Table S2). The particle diameters were 264­(±149; *n* = 206), 222­(±142; *n* = 260), and 195­(±97; *n* = 179) nm for Amolen, BlackMagic 3D, and ProtoPasta filaments, respectively; the differences between the means were all significant at the 95% confidence limit (2-tailed t-test). The particle density was 9 × 10^7^, 1 × 10^8^, and 3 × 10^8^ cm^–2^ for Amolen, BlackMagic, and ProtoPasta, respectively. Interestingly, the surface of each filament is not decorated uniformly with the Au NPs suggesting that the distribution of active sites for NP nucleation may be inhomogeneous. This observation is consistent with the hypothesis that the surface of the 3D-printed filament electrodes is not uniformly reactive. We quantified the total Au surface area by performing CV in a 2 M H_2_SO_4_ solution and integrating the oxide stripping peak at ∼0.9 V vs. SCE (Figure S4). The integrated charge was converted to the Au surface area assuming that 1 cm^2^ of Au required 0.390 mC to strip.^80^ The stripping data show that the ProtoPasta and Amolen samples had very similar Au surface areas, 7.1(±0.9) × 10^–3^ cm^2^ and 9(±3) × 10^–3^ cm^2^, respectively. The Au surface area on the BlackMagic 3D electrodes was slightly smaller 5.2(±0.9) × 10^–3^ cm^2^. The BlackMagic 3D Au surface area was significantly smaller than the Amolen at the 95% confidence limit (two-tailed Students t-test), while all other comparisons were similar.

After activating with NaOH (Figure 2iii, bottom row), the surface of the Amolen electrodes was largely unchanged, displaying a similar surface morphology as the polished sample. In contrast, the surfaces of both the BlackMagic and ProtoPasta electrodes increased in roughness. This increased roughness is likely caused by a combination of the saponification of the aliphatic ester groups in the PLA occurring at the polymer surface during electrolysis^62^ and etching/activation of the carbon black and graphene particles. Interestingly, each filament reacted differently to this etching procedure, with BlackMagic showing the largest increase in roughness, followed by ProtoPasta and Amolen. The trend in morphology change is consistent with the measured uncompensated resistance values (see below), which suggests that *iR*u losses may impact materials activation when using electrolysis.

#### Raman Spectroscopy Characterization

We performed Raman spectroscopy of each filament to characterize the defect levels and obtain structural information about the carbon particles in the composites (Figure 3).^81^ We note that we did not perform Raman measurements on the electrodes modified with Au NPs. Typically, the D band (≈1350 cm^–1^) and G band (≈1580 cm^–1^) are used to characterize graphitic carbons.^82^ The D band characterizes in-plane C–C stretching while the G band relates to out-of-plane vibrations.^82^ Figure 3a shows Raman spectra of alumina-polished ProtoPasta (blue trace), BlackMagic 3D (red trace), and Amolen (yellow trace) composite electrodes. Figure 3b shows the spectra of the polished and NaOH-activated electrodes. All spectra were normalized versus the height of the G band. The ProtoPasta and Amolen filaments have very broad peaks for both D and G bands, consistent with the carbon black additive, while the BlackMagic 3D filament has narrower bands, consistent with the graphene additive. Also evident on the BlackMagic 3D spectra are small D’ bands at ≈1620 cm^–1^. The ratio of the intensities of the D band and G bands (*I*_D_/*I*_G_) was used to provide an estimate of the defect sites, with higher values indicative of higher disorder. The *I*_D_/*I*_G_ ratios were 0.77, 0.61, and 0.72 for alumina-polished Amolen, BlackMagic 3D, and ProtoPasta, respectively, and 0.69, 0.54, and 0.72 for electrochemically activated Amolen, BlackMagic 3D, and ProtoPasta, respectively. Both of the carbon black-containing filaments (Amolen and ProtoPasta) show a higher degree of disorder, consistent with the carbon materials present in the composite. These data suggest that the carbon particles become less disordered after electrochemical activation in 0.5 M NaOH and shows that the electrochemical treatment has a significant impact on the carbon surfaces in addition to removing PLA. These *I*_D_/*I*_G_ ratios are comparable to previously reported results.^57,66^

**Figure 3:**
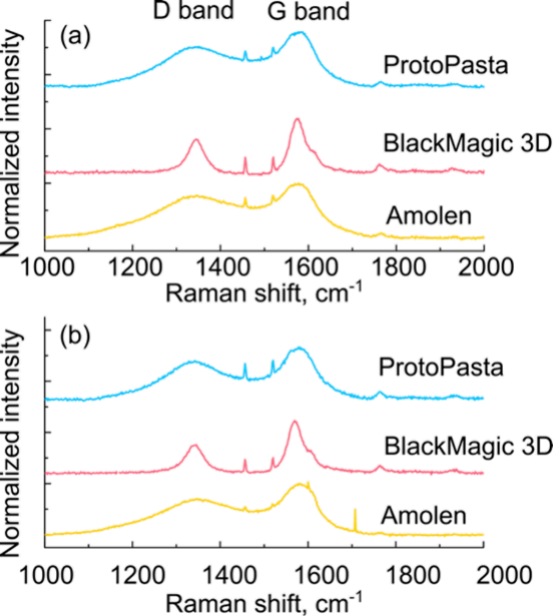
Raman spectroscopy characterization of Amolen, BlackMagic 3D, and ProtoPasta electrodes pretreated with (a) alumina polished and (b) polished and NaOH electrolysis. Raman measurements were performed with a 633 nm laser using 20× objective.

### Background Electrochemical Responses

#### Impact on Capacitance

The current of the polarizable region of a voltammogram is controlled by the capacitance of the electrode material. Capacitance is linearly correlated with background current, which in turn has a direct impact on detection limits. The double-layer capacitance of the three filaments was measured using the current difference of the forward and reverse scans in a cyclic voltammogram (eq 1) in 0.1 M KNO_3_. Example capacitance CVs are presented in Figure S5.

First, we compared how each filament reacted to the three pretreatments (Figure 4). For the polished samples, the capacitance increased in the order of ProtoPasta (2.3 ± 1.2 μF cm^–2^) ≈ Amolen (7.2 ± 1.7 μF cm^–2^) < BlackMagic (16 ± 3 μF cm^–2^). Using a similar electrolyte and measurement protocol, Hutton et al. reported the capacitance of boron doped diamond (BDD), glassy carbon (GC), and Pt electrodes to be 6.5±0.4, 24±2, and 35±3 μF cm^–2^, respectively.^83^ There were significant differences between the Blackmagic 3D and both the ProtoPasta (*p* < 0.001) and Amolen filaments (*p* < 0.01). No significant difference was observed between the ProtoPasta and Amolen (*p* = 0.09). For samples treated with electrolysis in 0.5 M NaOH, the capacitance order was ProtoPasta (1.2 ± 0.6 μF cm^–2^) ≈ Amolen (1.9 ± 0.9 μF cm^–2^) < BlackMagic (78 ± 28 μF cm^–2^). The increase in capacitance for the BlackMagic electrodes is consistent with the increased roughness observed in the SEM images in Figure 2. Again, significant differences were observed between BlackMagic 3D and ProtoPasta (*p* < 0.001) and Amolen (*p* < 0.001), but not between Amolen and ProtoPasta (*p* = 1). In the Au NP treated samples, the order was ProtoPasta (2.3 ± 0.7 μF cm^–2^) ≈ Amolen (2.6 ± 1.1 μF cm^–2^) < BlackMagic (9 ± 3 μF cm^–2^). We observed significant differences between BlackMagic 3D and both ProtoPasta (*p* < 0.01) and Amolen (*p* < 0.01), but not between Amolen and ProtoPasta. Interestingly, the capacitance of the ProtoPasta 3D filament was not affected by the pretreatment (*i.e.*, all the capacitance values were statistically similar). For the BlackMagic 3D, the capacitance of the NaOH electrolyzed filament was larger than the polished and Au NP samples (*p* < 0.05). For the Amolen, the capacitance of the polished Amolen filament was significantly larger than both the NaOH electrolyzed (*p* < 0.001) and the Au NP samples (*p* < 0.01).

**Figure 4:**
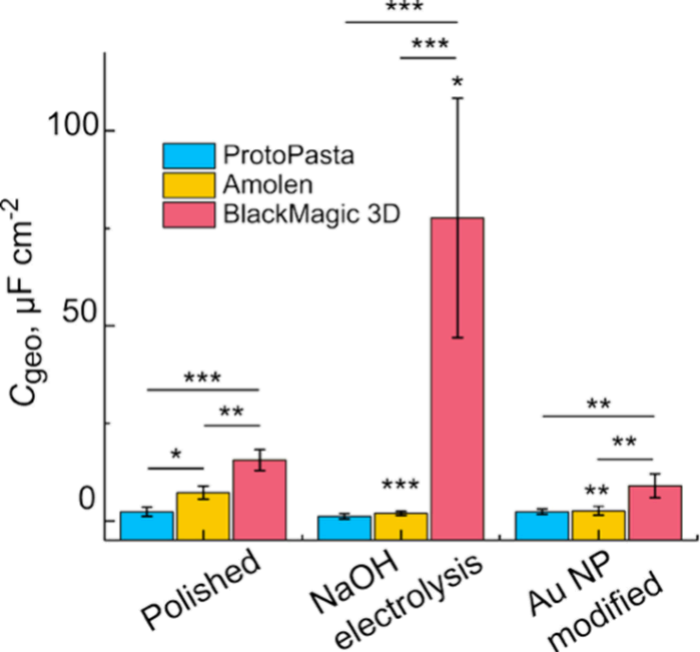
Comparison of geometric capacitance for the three filaments tested. Capacitance was measured using CV in 0.1 M KNO_3_ at 0.1 V s^–1^. ***: *p* < 0.001; **: *p* < 0.01; *: *p* < 0.05; n.s.: no significant difference; *n* = 3. Comparisons were made using one-way ANOVA using *post hoc* Bonferroni corrections.

#### Impact on Solvent Window

The solvent window is the potential range in which the material can perform Faradaic electrochemistry without being obscured by the oxidation/reduction of the solvent or supporting electrolyte.^75^ We determined the solvent window using the approach advocated by Hutton et al.:^83^

$$\mathrm{Solvent window}=E_{a}\left(j=+0.4{\mathrm{mA cm}}^{-2}\right)-E_{c}\left(j=-0.4{\mathrm{mA cm}}^{-2}\right)$$ 2

where *E*_a_ (*j* = +0.4 mA cm^–2^) is the potential where the current density (*j*) is +0.4 mA cm^–2^ and *E*_c_ (*j* = −0.4 mA cm^–2^) is the potential where the current density is −0.4 mA cm^–2^. Figure S6 shows representative solvent windows in deoxygenated 0.1 M KNO_3_ for ProtoPasta, BlackMagic 3D, and Amolen filament electrodes.

Figure 5 shows a statistical comparison of the solvent windows of the three materials. For the polished samples, the solvent windows rank from Amolen (3.8 ± 0.3 V) > ProtoPasta (3.55 ± 0.02 V) ≈ BlackMagic (3.28 ± 0.09 V), with a significant difference observed between the Amolen and BlackMagic samples (*p* < 0.05). After activating the electrodes by electrolysis in 0.5 M NaOH, the solvent windows of Amolen and ProtoPasta increased (4.0 ± 0.2 V and 3.87 ± 0.06 V, respectively) while the solvent window for BlackMagic 3D decreased (2.3 ± 0.3 V). The decrease in BlackMagic 3D solvent window was largely caused by an increased oxygen reduction reaction signal, as we have observed previously.^17^ There was no significant difference observed between the Amolen and ProtoPasta electrodes (*p* = 0.9). We observed significant differences between BlackMagic and both ProtoPasta (*p* < 0.001) and Amolen (*p* < 0.001). After modifying the electrodes with Au NPs, the solvent windows drastically decreased: Amolen (2.46 ± 0.10 V) ≈ ProtoPasta (2.46 ± 0.05 V) > BlackMagic (2.1 ± 0.2 V). There was a significant difference between the means of the BlackMagic and Amolen filaments (*p* < 0.05).

**Figure 5:**
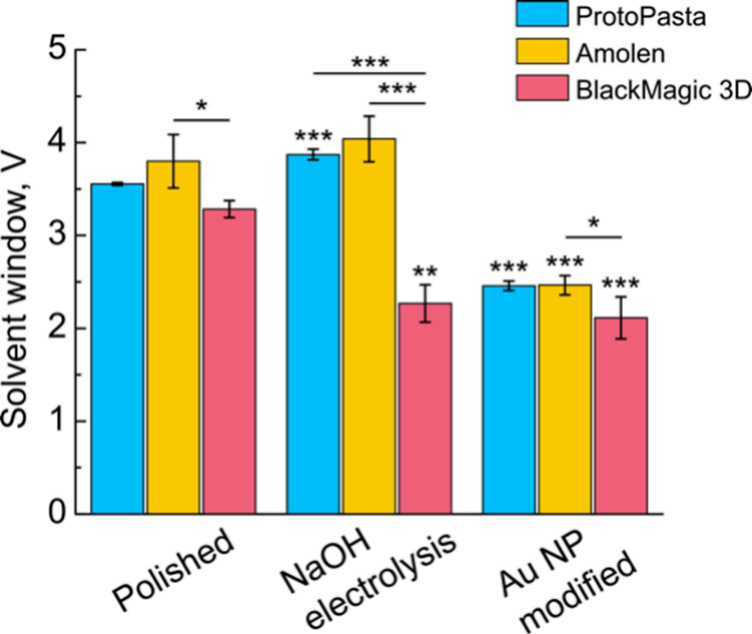
Comparison of solvent windows in 0.1 M KNO_3_ with a bar graph showing the mean ± standard deviation of the solvent windows for each filament. ***: *p* < 0.001; **: *p* < 0.01; *: *p* < 0.05; *n* = 3. Comparisons were made using one-way ANOVA using *post hoc* Bonferroni corrections.

The solvent windows of these materials compare extremely well with other carbon-based electrodes. Hutton et al. reported the solvent windows of various BDD electrodes, which are regarded as having the widest solvent windows of all carbon electrodes, in the range of 3.5–4.0 V, with higher solvent windows corresponding to lower electrochemical activity.^83^ We performed comparison experiments using GC and Au electrodes, but in both cases the current in the non-faradaic portions of the voltammogram was larger than the current threshold defined in eq 2 (Figure S7).

### Electrochemical Characterization with Outer-Sphere Redox Species

We first assessed the performance of each electrode to a representative outer-sphere redox reaction—the reduction of ruthenium­(III) hexamine (Ru­(NH_3_)_6_^3+^). The reduction of Ru­(NH_3_)_6_^3+^ is known to have fast heterogeneous electron transfer (HET) kinetics on a variety of carbon electrodes^84^ and is often used to assess the HET kinetics of 3D-printed electrodes. We performed a scan rate study using each filament pretreated by alumina polishing, electrodeposition of Au NPs, and electrolysis in NaOH to characterize mass transport, assess the quality of our electrode preparation method, and understand how Δ*E*p changes with pretreatment. We also performed these experiments using commercial GC and Au electrodes, as described in the Supporting Information.

Figure 6 shows representative CVs of Ru­(NH_3_)_6_^3+^ reduction at four scan rates (0.1, 0.25, 0.5, and 0.75 V s^–1^) for all nine conditions tested (three filaments pretreated three different ways). This range of scan rates was selected to assess if the current was diffusion-limited. The BlackMagic and ProtoPasta electrodes both demonstrate quasi-reversible, duck-shaped CVs under all conditions. The Amolen electrodes showed irreversible behavior for the polished samples, quasi-reversible behavior for the electrodeposited Au surface, and sigmoidal responses for the NaOH-treated samples. The data for the polished BlackMagic and ProtoPasta samples (Figure 6bi, 6ci) are qualitatively similar. The *i*_p_ versus *v* ^1/2^ plots for the reduction of Ru­(NH_3_)_6_^3+^ showed excellent linearity (*R* ^2^ > 0.99; Figure 6di) on these electrodes, demonstrating that the mass transport was controlled by semi-infinite linear diffusion of redox species to the electrode surface. For the polished Amolen electrodes, the CVs are distorted, with a more prominent reduction peak compared with the oxidation peak, as well as a slower post-peak current decay compared with BlackMagic and ProtoPasta. The distortion of the CV shape increases with increasing scan rate. Nevertheless, there is a strong correlation between *i*_p_ versus *v* ^1/2^ (*R* ^2^ > 0.96; Figure 6di).

**Figure 6:**
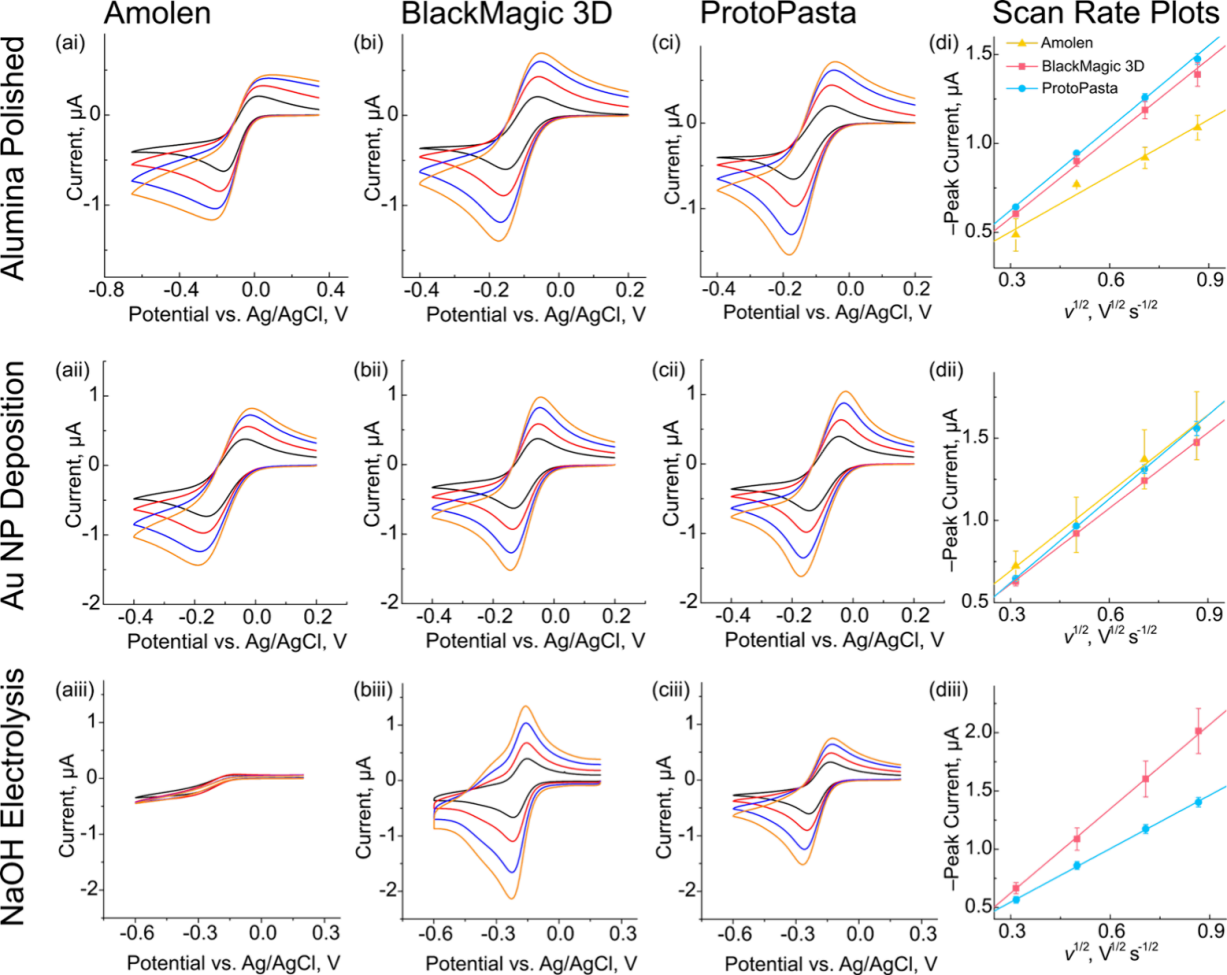
Effect of (i) alumina polishing, (ii) Au NP electrodeposition, and (iii) NaOH electrolysis on the outer-sphere reduction of Ru­(NH_3_)_6_^3+^ using (a) Amolen, (b) BlackMagic 3D, and (c) ProtoPasta filaments. Each electrode was measured using 0.1, 0.25, 0.5, and 0.75 V s^–1^. (d) Scan rate (Randles–Ševčík) plots for each electrode and pretreatment. Symbols in part d represent the mean of *n* ≥ 3 measurements, and error bars represent one standard deviation of the mean.

We compared the experimental slopes of the BlackMagic 3D and ProtoPasta electrodes to those calculated using the Randles–Ševčík equation for quasi-reversible electron transfer at 25 °C in Table S2:

$$i_{p}=2.63\times 10^{5}n^{3/2}AD^{1/2}c_{b}v^{1/2}$$ 3

where *n* is the number of electrons transferred (=1), *A* is the electrode area (cm^2^; from Table S1), *D* is the diffusion coefficient (=8.8 × 10^–6^ cm^2^ s^–1^),^85^ *c*_b_ is the concentration of Ru­(NH_3_)_6_^3+^ (=1 × 10^–6^ mol cm^–3^), *v* is the scan rate (in V s^–1^).^86^ Note that the numerical constant in quasi-reversible form of the Randles–Ševčík equation (=2.63 × 10^5^) is slightly smaller than the reversible form (=2.69 × 10^5^). For alumina-polished BlackMagic 3D and ProtoPasta electrodes, the experimental and theoretical values agree within the 95% confidence interval. We compared the experimental slope of the Amolen electrodes to the theoretical Randles–Ševčík slope for an irreversible reaction, given the large Δ*E*p of these samples (>200 mV, see below):

$$i_{p}=2.99\times 10^{5}\alpha^{1/2}n^{3/2}AD^{1/2}c_{b}v^{1/2}$$ 4

where α is the transfer coefficient (assumed to be 0.5) and all other variables have the same meanings as eq 3. The theoretical slope falls outside of the 95% confidence interval, suggesting there may be contributions from radial diffusion on the CV.

Figures 6aii, 6bii, and 6cii show the CVs for electrodes modified by electrodeposition of Au NPs. The presence of Au NPs was confirmed by electrochemical measurements in H_2_SO_4_ (Figure S4). In contrast to the polished samples, the CVs acquired using the samples modified by Au NP electrodeposition are all quasi-reversible. Additionally, the plots of *i*_p_ vs. *v* ^1/2^ for each of the filaments modified with Au agree reasonably well with the theoretical slope predicted by the Randles–Ševčík equation (<10% relative error for the BlackMagic 3D and ProtoPasta electrodes and ∼16% for the Amolen electrodes). While the deposition of Au NPs has very little impact on the diffusion response of the BlackMagic 3D and ProtoPasta electrodes, it completely changes the waveshape and diffusion characteristics of the Amolen sensors.

Figures 6aiii, 6biii, and 6ciii show CVs for electrodes pretreated by electrolysis in 0.5 M NaOH. Previous work has shown pretreatment of carbon/PLA electrodes improves electrochemical activity by two pathways: a chemical step, saponification of the aliphatic PLA esters exposing more carbon particles, and an electrochemical step, creation of new carbon–oxygen functional groups on the conductive particles.^20,62^ The Amolen electrodes exhibited microelectrode behavior, with sigmoidal voltammograms that are invariant to scan rate (Figure 6aiii). Previous theory and experiment show that macroscopic composite electrodes can exhibit microelectrode behavior when conductive particles are spaced far apart so that the radial diffusion layers to individual particles do not overlap.^87^ This preparation may represent an interesting future opportunity for sensor development given that the material behaves like an array of microelectrodes. Using BlackMagic 3D electrodes, we observed a second voltammetric feature at −0.4 V after NaOH electrolysis (Figure 6biii), suggesting a change in the surface chemistry of the carbon particles compared to the polished case. This peak reduces in magnitude after de-oxygenating the solution with Ar, but does not completely disappear (Figure S8). The peak currents and separations are nearly identical before and after purging with Ar, confirming that the presence of oxygen does not influence the overall data quality. In our previous work, we observed a similar feature on BlackMagic 3D electrodes.^17^ Another interesting feature of the BlackMagic 3D electrodes is that the Randles–Ševčík slope increased after NaOH electrolysis (Table S3). The effect of surface roughness on electrodes is typically small, becoming significant when the diffusion layer thickness is similar in size to the surface roughness. Using a combination of modeling and experiment, several groups have shown that electrode surfaces with μm-scale roughness behave identically to their planar counterparts at the scan rates employed herein.^88,89^ Because SEM is unable to provide a quantitative measure of surface roughness, we used AFM to characterize the roughness of polished and NaOH electrolyzed BlackMagic electrodes (Figure S9). The root mean square (RMS) roughness of the polished samples was 96 nm and was 204 nm for electrolyzed samples. The maximum roughness (*i.e.*, the distance between the lowest and highest spots in the images) also increased from 840 to 1928 nm for the polished and electrolyzed samples, respectively. Under the conditions of the CVs, the diffusion layer, δ (*≈* (*Dt*)^1/2^)*,* ranges from ∼12 to 50 μm, which is much larger than the measured roughness of the electrodes. In this intermediate region, where the roughness is approximately an order of magnitude smaller than the diffusion length at the highest scan rates, a mixed thin-layer/diffusion response is expected. While the Randles–Ševčík slopes of the NaOH electrolysis-treated BlackMagic electrodes showed excellent linearity (*R* ^2^ = 0.999), the slopes of log *i*p vs. log *v* plots had slopes of 0.55 suggesting a slight shift towards thin layer behavior (expected slope = 1 for pure thin layer), consistent with increased roughness. The NaOH electrolyzed BlackMagic electrodes do not experience a dramatic shift in Δ*E*p with scan rate (as would be expected for a system with moderate electron transfer kinetics and planar diffusion), increasing from 69±8 (0.1 V s^–1^) to 78±14 (0.75 V s^–1^) compared with 91±4 (0.1 V s^–1^) and 125±11 (0.75 V s^–1^) for polished samples. The ProtoPasta electrodes were largely unchanged after electrolysis in NaOH, displaying similar wave shapes, peak currents, and Randles–Ševčík slopes with alumina polishing and electrolysis in NaOH.

We can rationalize the responses of the electrodes to outer-sphere couples by considering their surfaces as partially blocked electrodes, where local active regions are surrounded by insulating regions. In these types of systems, which are analogous to randomly oriented micro- or nanoelectrode arrays, the size of the active locations, the spacing between the active locations, the kinetics of the electron transfer reaction, and the scan rate (*i.e.,* the time scale of the experiment) collectively influence the CV waveshape.^90^ The theory of these types of systems has been developed over the past several decades, first by Amatore et al.^91^ and subsequently by Compton and co-workers^92,93^ and then Amatore and co-workers.^94^ In the experiments described so far, the electron transfer reaction is outer-sphere and thus very fast, and so changes in the waveshape under each set of preparation conditions are largely determined by mass transport to the active sites. Critical to interpreting the responses of partially blocked electrodes is the relationship between the diffusion distance, δ, and the spacing between the electrode’s active sites, *d*. When δ ≫ *d*, the individual diffusion fields at the active sites coalesce to produce a collective linear diffusion field; when δ ≪ *d*, the individual diffusion fields do not overlap and the overall response is akin to that of an array of microelectrodes. In the two limiting cases, the voltammetry can be understood (and even modelled) by considering the array a uniform conductor or array of microelectrodes, respectively. In the intermediate region, where *δ* ≈ *d*, the diffusion fields partially overlap leading to complex voltammograms that can be misinterpreted.

The behavior experienced by the polished BlackMagic and ProtoPasta electrodes, all of the Au NP electrodes, and NaOH-treated ProtoPasta electrodes suggests that δ ≫ *d* in these cases. We make this assessment based on the close agreement between the experimental data and the Randles–Ševčík equation. The Amolen electrodes treated with NaOH suggest that δ ≪ *d*, because the voltammograms show steady state responses. The polished Amolen electrodes are likely in the intermediate region, where δ ≈ *d*, because of the distorted waveshapes of the polished Amolen CVs and the poor agreement between the experimental data and the Randles–Ševčík equation. The Amolen data suggests that the size of the carbon particles change during the NaOH electrolysis. Finally, electrolyzed BlackMagic demonstrates evidence of some surface confinement of the redox species, potentially caused by surface roughness and/or porosity.

We next analyzed the separation in anodic and cathodic peak potentials (Δ*E*p) at 0.1 V s^–1^ in order to understand how the pretreatments impact the shape of the CVs. We report the Δ*E*p instead of the standard HET rate constant (*k* ^0^) because determining the standard HET rate constant (*k* ^0^) using the Nicolson-Shain method is inappropriate when the circuit contains significant uncompensated resistance,^95,96^ as demonstrated by Meloni and co-workers using multi-physics simulation.^60^ While Δ*E*p and *k* ^0^ are related, the values for *k* ^0^ obtained with the Nicholson-Shain method would underestimate the true value of *k* ^0^ because of the large resistivity of the filaments. Additionally, the current density carried by each particle in a partially blocked electrode would be significantly higher than a uniform electrode, leading to complications interpreting kinetics.^90^

Figure 7 shows a bar graph comparing the average Δ*E*p values obtained on Amolen, BlackMagic 3D, and ProtoPasta electrodes. For the polished samples, the ProtoPasta (Δ*E*p = 108±11 mV) and BlackMagic (Δ*E*p = 91±4 mV) electrodes had the lowest Δ*E*p values, while the Amolen electrodes (Δ*E*p = 241±43) had significantly higher Δ*E*p values than BlackMagic (*p* < 0.01; *n* = 3) and ProtoPasta (*p* < 0.01; *n* = 3). We note that in this analysis, the interpretation of Δ*E*p for polished Amolen electrodes is complicated by the radial diffusion components discussed above. For samples pre-treated with electrolysis in NaOH, the BlackMagic 3D filament displayed the smallest Δ*E*p values, which approach reversibility (Δ*E*p = 69 ± 8 mV) and were statistically smaller than the ProtoPasta filaments (Δ*E*p = 96±14; *p* < 0.05). The lowered Δ*E*p values on BlackMagic electrodes are likely caused by increased roughness of the BlackMagic surfaces (Figure 2), causing changes in the mass transport behavior. The Amolen filaments pretreated using electrolysis in NaOH did not display diffusive CVs (Figure 6aiii). Au NP-modified electrodes prepared using ProtoPasta and BlackMagic 3D had statistically similar values for Δ*E*p (88±9 mV (pooled); *p* = 1) and both were significantly smaller than Amolen filaments (Δ*E*p = 166±41 mV; *p* < 0.05).

**Figure 7:**
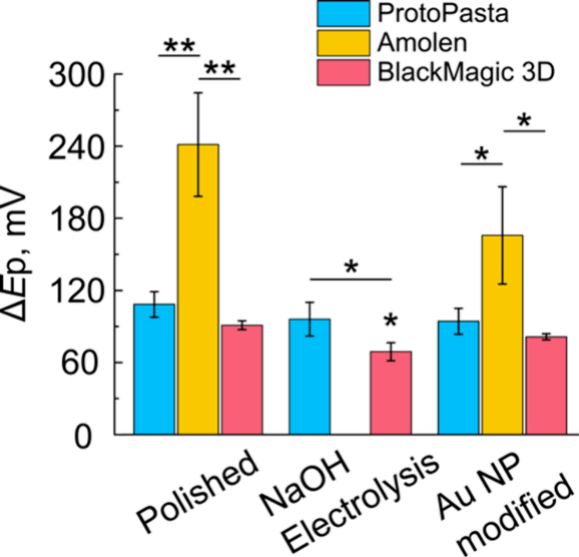
Peak potential separation of an outer-sphere redox reaction depend dramatically on the filament and pretreatment. Δ*E*p was determined using CV at 0.1 V s^–1^ using 1 mM Ru­(NH_3_)_6_^3+^ as an example outer-sphere redox mediator. Values represent the mean, and the error bars represent one standard deviation of *n* ≥ 3 independent samples. **p < 0.01; *: *p* < 0.05; comparisons were made using one-way ANOVA using *post hoc* Bonferroni corrections.

These data can also be used to understand how the Δ*E*p properties of a filament change with the pretreatments. ProtoPasta filament has statistically similar (*p* > 0.05; *n* = 3) values of Δ*E*p for all three pretreatments (=100 ± 12 mV for the pooled data). This indicates that Δ*E*p values on ProtoPasta are limited by the resistivity of the polymer rather than the HET rate. The Δ*E*p values for BlackMagic 3D were significantly smaller for the NaOH electrolysis samples than the polished samples (*p* < 0.05; *n* = 3); however, the Au NP were statistically similar to the polished samples. As mentioned above, the lower Δ*E*p is likely caused by changing mass transport conditions observed with BlackMagic electrodes pretreated with NaOH electrolysis. While BlackMagic 3D and ProtoPasta give similar responses regardless of pretreatment, the Amolen electrodes behaved dramatically different depending on the pretreatment. The polished Amolen displays very broad Δ*E*p (=241±43 mV). After NaOH electrolysis, no peaks were measured for any of the Amolen samples prepared, instead showing steady-state responses. However, the electrodeposition of Au NPs yielded a moderate improvement in kinetics (Δ*E*p = 166±41 mV; *p* > 0.05).

We measured uncompensated resistance (*R*u) of these electrodes using electrochemical impedance spectroscopy (EIS) as described in Section 5 of the Supporting Information. Representative Nyquist plots are shown in Figure S10. The data were fit to a simple Randles circuit without diffusion and the *R*u values were extracted from the fit. The Amolen, BlackMagic 3D, and ProtoPasta electrodes had values of 18±3, 1.4±0.4, and 3±1 kΩ, respectively. The trend observed in the *R*u data matches the trends observed in the Δ*E*p data for Ru­(NH_3_)_6_^3+^ reduction (Amolen > ProtoPasta ≈ BlackMagic 3D), supporting our hypothesis that the changes in Δ*E*p are largely caused by the resistivity of the material.

We repeated the characterization of outer-sphere redox species using the oxidation of FcMeOH to test if the ruthenium­(III) hexaamine’s redox potential^97^ impacted the rate analysis (summarized in Figure S11). The results obtained for FcMeOH oxidation were statistically similar to Ru­(NH_3_)_6_^3+^ reduction (Figure S12).

Taken together, these data highlight the following: first, understanding the response of 3D-printed filament electrodes necessitates the use of partially blocked electrode theory^90^ and considering the mass transfer changes caused by surface roughness. The subtle effects of mass transfer to micro-/nanoscale active sites embedded within an insulating matrix are most apparent with the Amolen electrode, where dramatic changes in the voltammetry are observed with each pre-treatment. These changes could easily be mistaken as changes in electron transfer kinetics (or other effects) without careful consideration of the experimental setup and conditions. Second, the data reinforce the need to compare any pretreatment results with theory (*e.g.*, Randles–Ševčík) to draw any conclusions about the behavior of the materials. The insights about the Amolen filament described above are only possible because there is a rather large discrepancy in the experimental Randles–Ševčík data with the theory, and this comparison is only possible because a polished electrode with a well-defined geometry was used as a control. Third, the impact of pretreatment on ProtoPasta and BlackMagic is minimal. The only pretreatment that yielded significant changes to Δ*E*p of either filament was NaOH electrolysis on the BlackMagic 3D electrode; the changes to NaOH-treated BlackMagic are likely caused by mass transport changes due to increased roughness and not changes in the electron transfer kinetics. The consistency of Δ*E*p values across the pre-treatments, along with the *R*u data, clearly show that the major determining factor for the response of these electrodes to outer-sphere species is the *R*u. This demonstrates that for these materials, it is critically important to use an experimental configuration that minimizes uncompensated resistance losses (*e.g.*, small electrodes with short connection pathways and low contact resistance), such as the configuration described herein.

### Electrochemical Characterization with Inner-Sphere Redox Species

We next studied a series of inner-sphere redox mediators which were surface sensitive (Fe­(CN)_6_^4–^ oxidation; Figure 8i), sensitive to the presence of surface oxides (Fe^2+^ oxidation; Figure 8ii), and require adsorption for rapid electron transfer (dopamine oxidation; Figure 8iii) using the three filaments and three pre-treatments described above. These three redox mediators were selected because they broadly encompass the types of inner-sphere interactions relevant analytes will experience at the electrode surface. We used these data to assess the HET kinetics (via the Δ*E*p) and performed a statistical comparison of these values in Figure 8d.

**Figure 8:**
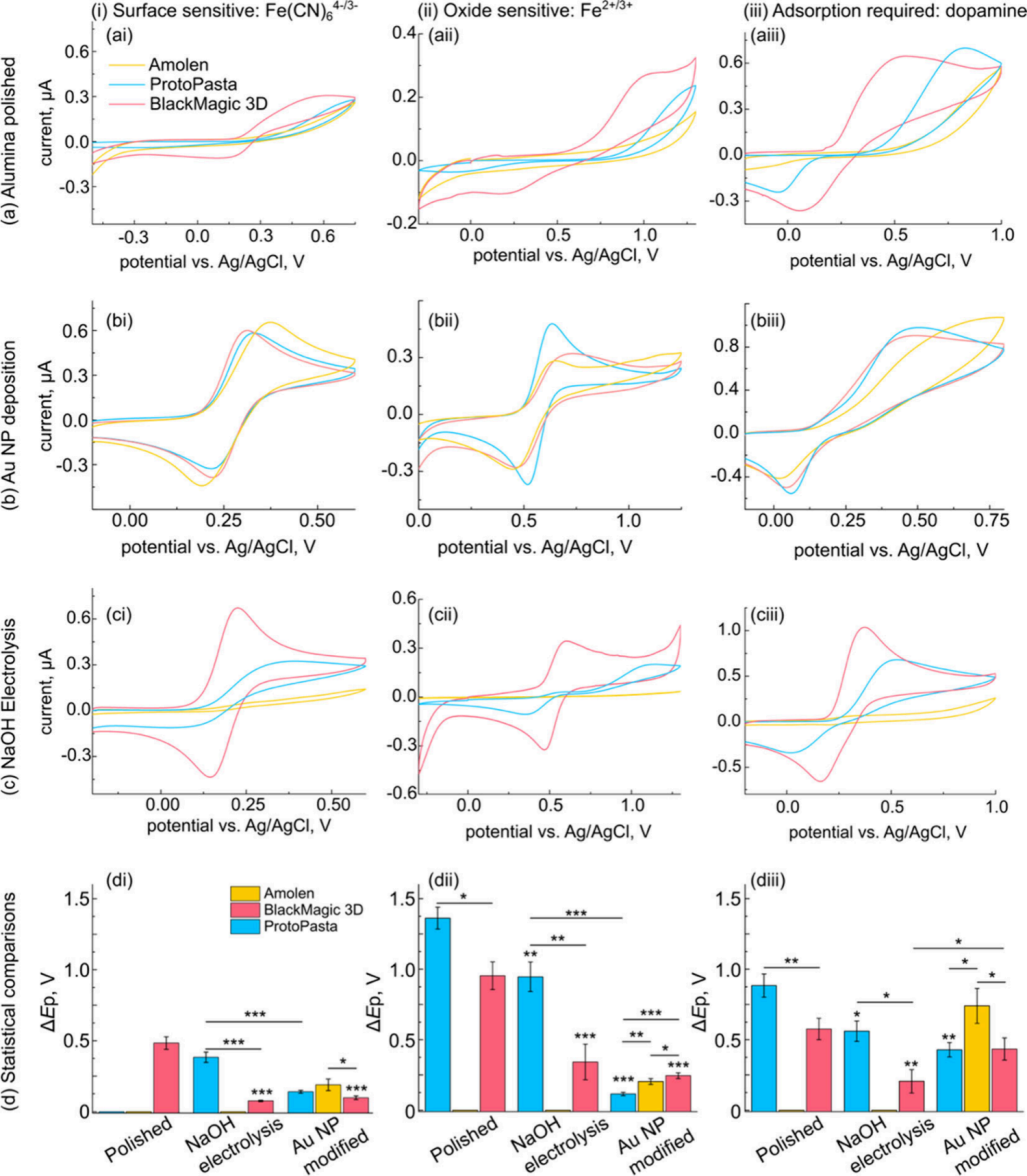
Each filament responds differently to each pretreatment when probing inner-sphere electron transfer reactions. The effect of (a) alumina polishing, (b) Au NP electrodeposition, and (c) NaOH electrolysis on Amolen (yellow), BlackMagic 3D (red), and ProtoPasta (blue) filaments for the inner-sphere redox reactions of (i) Fe­(CN)_6_^4–^ oxidation, (ii) Fe^2+^ oxidation, and (iii) dopamine oxidation. (d) Statistical comparison of mean Δ*E*p data obtained for the redox species listed above. Data are presented as the mean of at least three measurements performed on separately prepared electrodes; the error bars represent one standard deviation of the mean. ***: *p* < 0.001; **: *p* < 0.01; *: *p* < 0.05; *n* = 3. Comparisons were made using one-way ANOVA using *post hoc* Bonferroni corrections. Electrolytes and experimental conditions: (i) 1.5 mM Fe­(CN)_6_^4–^ in 0.1 M KNO_3_; *v* = 0.1 V s^–1^; (ii) 1 mM Fe^2+^ in 0.1 M HClO_4_, *v* = 0.1 V s^–1^; (iii) 1 mM dopamine hydrochloride in 1X PBS, *v* = 0.25 V s^–1^.

#### Surface Sensitive Probe: Fe­(CN)_6_^4–^ Oxidation

We first investigated Fe­(CN)_6_^4–^ oxidation, which is an inner-sphere redox reaction that is sensitive to both the electrode surface condition and area (Figure 8i, left column).^81^ For the polished samples (Figure 8ai), BlackMagic 3D was the only filament to present peaks within the tested potential window (−0.5 to 0.75 V); however, these peaks were spaced very far apart (Δ*E*p = 483±44 mV), suggestive of sluggish electron transfer. After electrodepositing Au NPs, all three filaments had diffusion-limited responses that had peak separations of 189±41, 140±10, and 98±13 mV for Amolen, ProtoPasta, and BlackMagic 3D, respectively (Figure 8bi). As shown in Figure 8di, the ProtoPasta had statistically similar responses to both the Amolen and BlackMagic 3D filaments (*p* > 0.05; *n* ≥ 3); the behavior of the Amolen filament was statistically larger than the BlackMagic 3D (*p* < 0.05; *n ≥* 3). After electrolysis in NaOH, the Amolen filament did not display a diffusive CV, while the BlackMagic 3D and ProtoPasta filaments did (Figure 8ci). The BlackMagic filament improved after NaOH activation (Δ*E*p = 76±5 mV), achieving a similar peak separation to the outer-sphere couples (*ΔE*p = 69±8). However, the ProtoPasta electrode was sluggish (Δ*E*p = 383±36 mV), which is significantly broader than Ru­(NH_3_)_6_^3+^ (Δ*E*p = 96±14 mV). The Δ*E*p of the BlackMagic 3D electrode was statistically smaller than the ProtoPasta electrode (*p* < 0.001; Figure 8di). A comparison of these two values highlights the advantages of using graphene as the conductive particles, where the surfaces are more active towards outer-sphere couples. For the BlackMagic electrodes, both electrolysis in NaOH and electrodeposition of Au NPs statistically improved the response compared with the polished samples (*p* < 0.001; *n* = 3). For ProtoPasta electrodes, the electrodeposition of Au NPs statistically improved the response compared with the NaOH electrolysis (*p* < 0.001; *n* = 3). For Amolen, the only pretreatment that yielded quasi-reversible electrochemical data was the electrodeposition of Au NPs. Commercial GC, pretreated with alumina polishing, and Au, pretreated by cycling in acid, electrodes gave Δ*E*p values of 268 and 110 mV, respectively (Figure S14).

#### Oxide Sensitive Probe: Fe^2+^ Oxidation

We next investigated the inner-sphere oxidation of Fe^2+^, which has been previously shown to be sensitive to surface oxides – particularly carbonyl groups.^50,81,98^ The polished samples yielded poor responses for all electrodes. The BlackMagic and ProtoPasta electrodes displayed broad, irreversible CV peaks (Δ*E*p = 954±98 and 1362±77 for BlackMagic and ProtoPasta, respectively; Figure 8aii). The Δ*E*p values of BlackMagic electrodes were statistically smaller than the ProtoPasta electrodes (*p* < 0.05; *n* = 3), as shown in Figure 8dii. All electrodes prepared with Au NPs displayed diffusion-limited CVs with moderate peak separations (Figure 8bii). The ProtoPasta filament showed the smallest peak separation (Δ*E*p = 115±12 mV) that was statistically smaller than both the BlackMagic (Δ*E*p = 245±20 mV; p < 0.001) and Amolen (Δ*E*p = 204±21 mV; *p* < 0.01) electrodes. Electrodes pretreated with electrolysis in NaOH showed varied responses (Figure 8cii). The BlackMagic electrodes displayed improved peak separations (Δ*E*p = 342±126 mV), the ProtoPasta electrodes showed broad peaks (Δ*E*p = 946±105 mV), and the Amolen electrodes were not responsive. Similarly, the polished BlackMagic electrodes displayed smaller peak separations than the polished ProtoPasta electrodes (*p* < 0.01; *n* = 3). For BlackMagic electrodes, both Au NP electrodeposition and NaOH electrolysis showed improved, quasi-reversible responses compared with polished samples (*p* < 0.001; *n* = 3). For ProtoPasta electrodes, the NaOH-treated samples were irreversible, but had faster kinetics compared with the polished samples (*p* < 0.01; n = 3). However, the Au NP ProtoPasta samples showed the fastest kinetics of all tested conditions and were statistically faster than the polished and NaOH-treated samples (*p* < 0.001; *n* = 3). Similar to the Fe­(CN)_6_^4–^ data, the only pretreatment that yielded detectable peaks for Amolen electrodes was the electrodeposition of Au NPs. Commercial GC, pretreated with alumina polishing, and Au, pretreated by cycling in acid, electrodes gave Δ*E*p values of 267 and 87 mV, respectively (Figure S14).

#### Adsorption Required Probe: Dopamine Oxidation

The final outer-sphere redox reaction we tested was dopamine oxidation, which requires adsorption onto the carbon surface to have facile HET kinetics.^81,99^ The oxidation kinetics depend on the surface quality of the carbon, as well as the carbon allotrope.^100^ The polished BlackMagic and ProtoPasta electrodes showed poor responses with large peak separations (Figure 8aiii). The Δ*E*p of the BlackMagic (=576±76 mV) electrodes were statistically smaller than the ProtoPasta electrodes (=884±82 mV; *p* < 0.01; *n* = 3). For the electrodeposited Au NP samples, all three electrode materials displayed relatively poor responses (ΔEp > 400 mV). The Amolen Au NP electrodes were statistically slower than both the BlackMagic (*p* < 0.05; *n* ≥ 3) and the ProtoPasta electrodes (*p* < 0.01; *n* ≥ 3). For samples pretreated with NaOH electrolysis, the BlackMagic 3D electrodes displayed quasi-reversible responses (Δ*E*p = 205 ± 83 mV), the ProtoPasta samples showed irreversible behavior (Δ*E*p = 561±72), and the Amolen electrodes did not have peak-shaped responses. Comparing the three pre-treatments on BlackMagic electrodes shows that only the NaOH electrolysis yielded improved responses compared with the polished samples (*p* < 0.01; *n* = 3). For ProtoPasta electrodes, both the Au NP (*p* < 0.01; *n* = 3) and NaOH samples (*p* < 0.05; *n* = 3) had improved kinetics in comparison with the polished electrodes. For the Amolen electrodes, only the Au NP-modified samples showed any redox activity. Commercial GC, pretreated with alumina polishing, and Au, pretreated by cycling in acid, electrodes gave ΔEp values of 189 and 82 mV, respectively (Figure S14).

### Summary of Electrochemical Responses

Table 2 summarizes the results of the electrochemical characterizations presented above with the goal highlighting some interesting features of the results. First, the Amolen electrodes, which have been previously screened and discarded based on their sub-par performance,^29,101^ demonstrate interesting and unique responses to the three pretreatments. We observed three unique responses that were explained using partially blocked electrode theory. Second, BlackMagic 3D and ProtoPasta are statistically similar when polished with alumina or with electrodeposited Au NPs for outer-sphere redox reactions. The similar *R*u values of the BlackMagic 3D and ProtoPasta of the electrodes used in the configuration described here supports the hypothesis that *R*u is the major factor in determining Δ*E*p. Third, BlackMagic 3D electrodes show strong responses for inner-sphere electrochemistry. BlackMagic 3D electrodes activated with NaOH electrolysis showed the smallest Δ*E*p values (and largest peak currents) for every inner-sphere redox species studied. These responses are not entirely down to improvements in HET kinetics, but rather a combination of changing mass transport conditions and faster electron transfer. The ProtoPasta electrodes showed mixed responses towards inner-sphere couples, with the best results obtained after depositing Au NPs. This suggests that conductive graphene is a much better choice than carbon black for electrochemical sensing applications, and highlights the advantages of using electrodeposited metals for creating high-quality electrodes using 3D printing. The BlackMagic electrodes lost most of the PLA mass in TGA at the lowest temperature and showed very little/no additive peak. Fourth, the background responses of BlackMagic 3D electrodes are considerably larger than the Amolen or ProtoPasta electrodes. For sensing, background signals are critically important because they have a direct influence on the signal-to-noise ratio, with lower capacitance improving signal-to-noise. The capacitance of BlackMagic 3D is ∼4 to 30x larger than ProtoPasta, depending on the pretreatment, and the solvent windows are smaller by ∼0.3 V. These are likely caused by changes brought on by the inherent characteristics of the graphene particles (*e.g.*, the density of states), the surface chemistry of the graphene after activation compared with carbon black, and the number of exposed particles. Collectively, these factors lead to increased capacitance and smaller solvent windows caused by faster kinetics for hydrogen and oxygen evolution.

**Table 2:** Summary of the Electrochemical Characterization Data from Results and Discussion ^a^

	Amolen			BlackMagic 3D			ProtoPasta		
	Polished	Electrolysis	Au NPs	Polished	Electrolysis	Au NPs	Polished	Electrolysis	Au NPs
Capacitance, μF cm^–2^	7.2 ± 1.7	1.9 ± 0.9	2.6 ± 1.1	16 ± 3	78 ± 28	9 ± 3	2.3 ± 1.2	1.2 ± 0.6	2.3 ± 0.7
Solvent window, V	3.8 ± 0.3	4.0 ± 0.2	2.46 ± 0.10	3.28 ± 0.09	2.3 ± 0.3	2.1 ± 0.2	3.55 ± 0.02	3.87 ± 0.06	2.46 ± 0.05
Ru(NH_3_)_6_^3+^	241 ± 43	n.p.	166 ± 41	91 ± 4	69 ± 8	81 ± 3	108 ± 11	96 ± 14	94 ± 11
Fe(CN)_6_^4–^	n.p.	n.p.	189 ± 41	483 ± 44	76±5	98 ± 13	n.d.	383 ± 36	140 ± 10
Fe^2+^	n.p.	n.p.	204 ± 21	954 ± 98	342 ± 126	245 ± 20	1362 ± 77	946 ± 105	115 ± 12
Dopamine	n.p.	n.p.	740 ± 124	576 ± 76	205 ± 83	434 ± 78	884 ± 82	561 ± 72	429 ± 51

a n.p.: Voltammograms did not produce peaks within the potential scan window. Note that the Δ*E*p values for Ru­(NH_3_)_6_^3+^, Fe­(CN)_6_^4–^, and Fe^2+^ were measured at 0.1 V s^–1^; the ΔEp values for dopamine were measured at 0.25 V s^–1^.

## Conclusions

In this study, we investigated how the identity and pretreatment of the 3D-printed filament impact the electrochemistry relevant to electrochemical sensing applications. We systematically examined the factors that control the electrochemical properties of commercial carbon thermoplastic 3D-printed filament electrodes, focusing on three commercially available filaments and three representative pretreatments that have previously been used for high-quality sensing applications. Although each of these filaments and pre-treatments have been examined individually in prior studies, our work offers the first systematic comparison across multiple filaments and pretreatments under identical conditions. This approach reveals important distinctions in electrochemical behavior that would remain hidden in isolated investigations. We evaluated each pretreatment with each filament by performing physical (SEM, Raman) and electrochemical (capacitance, solvent windows, and CVs using outer- and inner-sphere redox mediators) methods. The evidence present supports the following insights.

*The heterogeneity of the electrodes must be considered when characterizing electrochemical properties.* The electrochemical reactivity of 3D-printed filament electrodes is very likely heterogeneous, with active particles suspended in an insulating matrix. Therefore, in order to correctly analyze the responses of these electrodes partially blocked electrode theory should be employed.^90^ The subtle effects of mass transfer to micro-/nanoscale active sites embedded within an insulating matrix are most apparent with the Amolen electrode, where dramatic changes in the voltammetry are observed with each pre-treatment. These changes could easily be mistaken as changes in electron transfer kinetics (or other effects) without careful consideration of the experimental setup and conditions. The data also reinforce the need to compare experimental results with theory (*e.g.*, Randles–Ševčík) to draw meaningful conclusions about the behavior of the materials. The insights about the Amolen filament described above are only possible because there is a rather large discrepancy in the experimental Randles–Ševčík data with the theory, and this comparison is only possible because a polished electrode with a well-defined geometry was used.

*The electrode form factor matters when evaluating a 3D printable material’s electrochemical properties because uncompensated resistance can lead to misinterpretations of the HET kinetics.* 3D-printed electrodes typically have broad peak separations (Δ*E*p), often attributed to slow kinetics, even for fast, outer-sphere redox reactions like the reduction of Ru­(NH_3_)_6_^3+^. Using a combination of experiment and finite element modeling, Meloni and co-workers recently showed that the relatively poor responses can be largely attributed to resistance losses due to contact resistance as well as in the material (caused by the relatively low conductivity of the filaments).^60^ Importantly, they also conclusively showed that it is impossible to disentangle slow kinetics from high uncompensated resistance in a single voltammetric experiment.^60^ In the majority of previous studies, the electrodes range in diameter from 3–6 mm, which draw significantly larger currents (*e.g.*, 10s to 100s of μA) than the electrodes employed here. The results obtained here support Meloni and co-workers’ study, showing that the combination of a relatively small macroelectrode (*d* ≈ 400–600 μm) and short connection pathway (< 1 cm) leads to significantly decreased uncompensated resistance losses and relatively low currents, enabling fairly low peak separations for Ru­(NH_3_)_6_^3+^ and FcMeOH. The mean Δ*E*p values for Au NP modified BlackMagic 3D electrodes was 81±3 mV, with the best-performing samples achieving a peak separation of 79 mV (at 0.1 V s^–1^; Figure 5). These are among the smallest recorded Δ*E*p values for electrodes fabricated from conductive filaments, demonstrating the importance of our approach. Our measurements support the hypothesis that uncompensated resistance is the determining factor in establishing the peak separations for outer-sphere redox processes. In the cases of both BlackMagic 3D and ProtoPasta, the various pretreatments did not show statistically significant differences in Δ*E*p, with the exception of electrolysis-treated BlackMagic 3D. Looking forward, we expect that with further miniaturization^102^ and optimization of the contact resistance, it should be possible to achieve reversible responses for 3D-printed materials.

*The selected filaments do not respond to pre-treatments identically, and therefore detailed characterization (especially electrochemical characterization, including non-faradaic background processes) must be employed when developing new customized filaments.* It is widespread in the literature to perform a single pre-treatment on a 3D-printed electrode to evaluate the effectiveness of the material. The data in Table 2 clearly show that each filament responds uniquely to the three pretreatments studied, and future studies should study several representative pretreatment options when characterizing new/existing materials to properly evaluate the usefulness of a filament for a specific application. Our data show the least amount of variation for samples pretreated with electrodeposited Au NPs. It is also worth noting that using outer-sphere mediators is useful when evaluating a pretreatment, as these experiments primarily inform on the uncompensated resistance. It is critical to have an understanding of this behavior before moving on to more advanced redox reactions. We note that multiple redox mediators should be probed in order to provide a more complete understanding of the interface once the outer-sphere behavior is understood. Like other carbon electrode materials,^75^ we envision that optimizing 3D-printed composite electrodes for specific sensing applications will require targeted pretreatment protocols along with detailed studies of both the chemical and physical properties of the interfaces.^17^

*Electrodepositing metal nanoparticles is a very effective method of producing high-quality sensing interfaces regardless of filament and is probably underutilized in the field.* While some studies have used electrodeposited metal nanoparticles for improving the behavior of 3D-printed electrodes (*e.g.*, refs 8,25,60 ) it is less common than NaOH electrolysis, solvent treatments, or laser activation. Based on the results obtained here, electrodeposited Au nanoparticles display very promising behavior, even on filaments that have non-ideal responses under almost every other circumstance.

## Supplementary Material



## Notes

(1) Image analysis of prepared electrodes, (2) Au electrodeposition preparation and characterization, (3) cyclic voltammograms of 0.1 M KNO_3_ for solvent window and geometric capacitance analysis, (4) additional data for the analysis of Ru­(NH_3_)_6_^3+^ reduction on alumina polished electrodes, (5) atomic force microscopy (AFM) images of BlackMagic 3D electrodes, (6) electrochemical impedance spectroscopy of polished electrodes, (7) outer-sphere ET measurements using FcMeOH, (8) CV measurements performed with commercial GC and Au disk electrodes (PDF)
