# Supplementary material for: A Comparative Study of Conductive 3D Printing Filaments for Electrochemical Sensing Applications Pretreated by Alumina Polishing, Electrochemical Activation, and Electrodeposition of Au Nanoparticles
Source: ACS Electrochem. 2025 Oct 28;1(11):2386–401. doi: 10.1021/acselectrochem.5c00240 (PMC12598703; doi:10.1021/acselectrochem.5c00240)
Supplement: Supplementary file 1 [file ec5c00240_si_001.pdf]

Electronic supporting information for:

A comparative study of conductive 3D printing filaments for electrochemical sensing applications pretreated by alumina polishing, electrochemical activation, and electrodeposition of Au nanoparticles

Shakir Ahmed,[†a] Enock G. Arthur,[†b] Tanner Obrzut,[†a] Ricoveer Shergill,[b,c] Alexa Williams,[a] Kelvin Wamalwa,[a] Zackary D. Epright,[a] Cameron Darvish,[a] Yousef Khatib,[a] Wanlu Li,[a] Bhavik A. Patel,[b,c] and Glen D. O'Neil [a,d*]

[a.] Department of Chemistry and Biochemistry, Montclair State University, Montclair, NJ 07043, United States

[b.] School of Applied Sciences, University of Brighton, Brighton, BN2 4GJ, UK

[c.] Centre for Lifelong Health, University of Brighton, Brighton, BN2 4GJ, UK

[d.] Sokol Institute for Pharmaceutical Life Sciences, Montclair State University, Montclair, NJ 07043, United States

Email: oneilg@montclair.edu

[†] These authors contributed equally

Section **Page**

S1. Image analysis of prepared electrodes…………………………………………………………….. S2

S2. Au electrodeposition: preparation and characterization ……………………………………………... S3

S3. Cyclic voltammograms of 0.1 M KNO_3 for solvent window and geometric capacitance analysis…. S5

S4. Additional data for the analysis of $Ru(NH_3)_6^{3+}$ reduction on alumina polished electrodes………….. S7

S5. Atomic force microscopy (AFM) images of BlackMagic 3D electrodes……………………………. S8

S6. Electrochemical impedance spectroscopy of polished electrodes……………………………………S9

S7. Outer-sphere ET measurements using ferrocene methanol (FcMeOH)…………………………… S10

S8. Experiments performed with commercial glassy carbon and Au electrodes……….……………… S11

S1. Image analysis of prepared electrodes

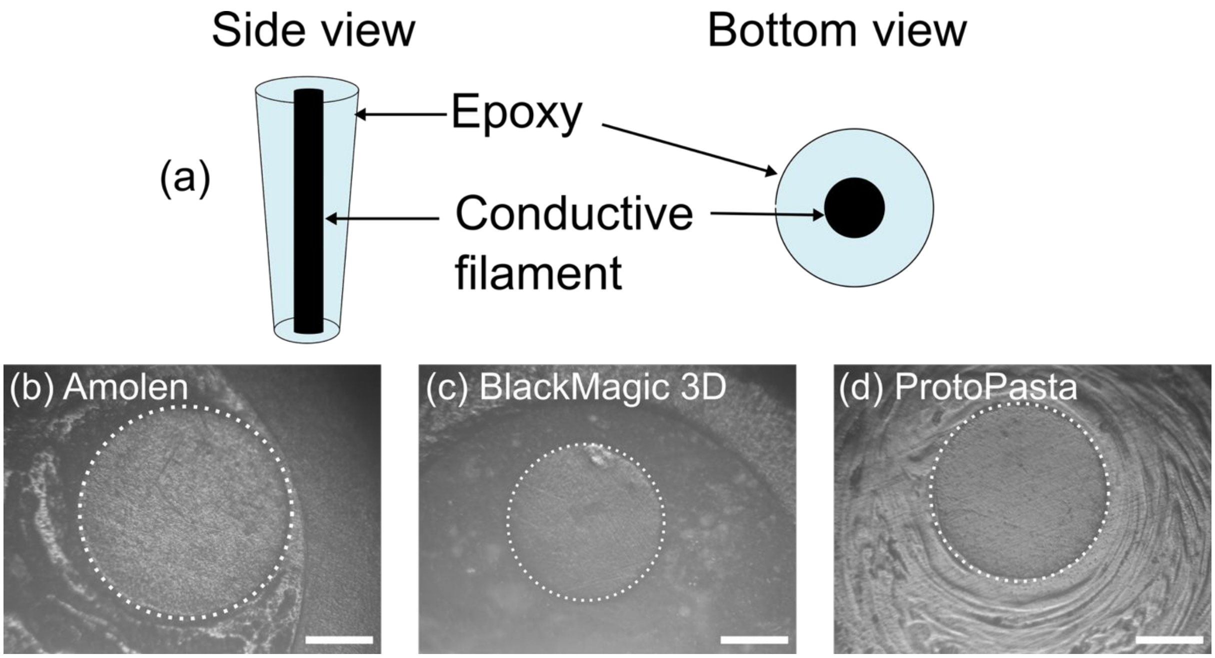

Figure S1. Schematic of electrode geometry and representative images of polished electrodes taken after fabrication. The dotted outline serves as a guide to the eye for the electrode material. The scale bars represent 200 µm.

Table S1. The measured areas and diameters of electrodes that were used in this study. Values reported as the mean ± one standard deviation of the mean for *n* = 3 independently prepared electrodes.

Filament	Area, cm^2	Diameter, cm
Amolen	0.0030 ± 0.0002	0.062 ± 0.016
BlackMagic 3D	0.00181 ± 0.00008	0.048 ± 0.010
ProtoPasta	0.00199 ± 0.00002	0.050 ± 0.005

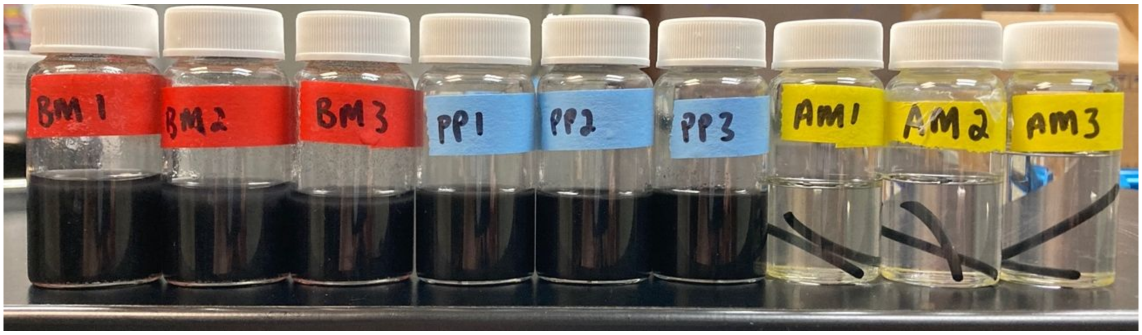

Figure S2. Images of filaments after spending 36 hours in THF. Each vial contains ~10 mL of solvent and two ~2 cm sections of filament. BM: BlackMagic 3D; PP: ProtoPasta; AM: Amolen.

S2. Au electrodeposition: preparation and characterization

Au nanoparticles were deposited on 3D-printed electrode surfaces as described in the main text. Briefly, in a 2 mM H_2AuCl_6 and 0.1 M HCl solution, a –1 V pulse was applied for two seconds followed by a lower –0.4 V pulse for 18 seconds. During the first two seconds of the deposition, the current is noisy and difficult to interpret because several reactions are occurring simultaneously: Au electrodeposition, oxygen reduction, and hydrogen evolution. A representative *i-t* curve for the deposition is shown in Figure S3 below.

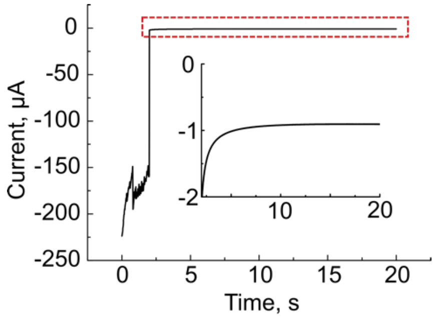

Figure S3. Representative Au NP deposition chronoamperometry traces that were recorded on a polished BlackMagic 3D electrode. The inset shows a close-up of the highlighted box.

We estimated the amount of Au on the surface by measuring Au oxidation and stripping in 0.5 M H_2SO_4 (Figure S4). Clear oxidation (~1.2-1.5 V) and stripping (~0.9 V) were observed after Au NP electrodeposition for each filament. The Au stripping peak was integrated, and the charge passed was converted to surface area using the well-known conversion factor (=0.390 mC cm^{-2}). Table S2 shows the average Au surface areas obtained for multiple samples.

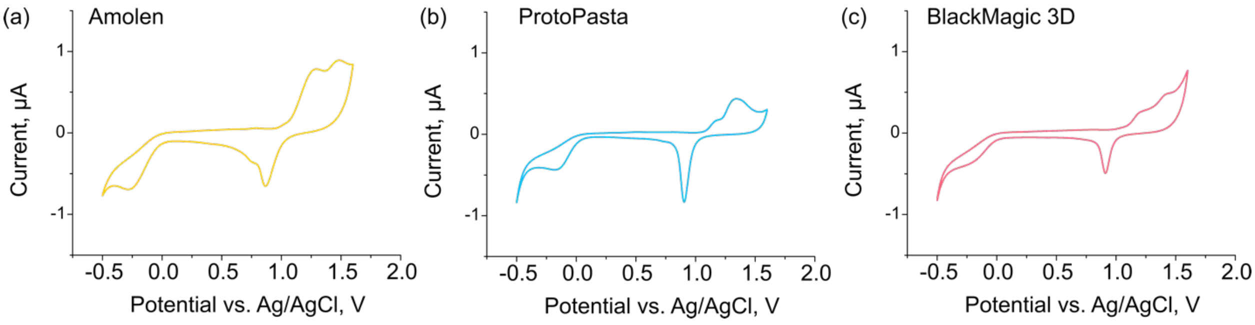

Figure S4. Representative CVs showing Au NP stripping peaks for (a) Amolen, (b) ProtoPasta, and (c) BlackMagic 3D electrodes.

Scanning electron microscopy (SEM) images were used to characterize the density and diameter of electrodeposited Au NP. The SEM images in Figure 1aii, 1bii, and 1cii in the main text were analyzed using ImageJ by first thresholding the raw image and performing a particle analysis (both are standard operations in ImageJ). The density was estimated from the NP count of a representative portion of the

image divided by the area of the portion in cm^2. The results of this microscopy characterization are presented in Table S1.

Table S2. Summary of Au NP characterization using electrochemistry and electron microscopy. Values are represented as the mean ± one standard deviation.

	**Au surface area, ×10^{-3} cm^2**	**Au NP density, cm^{-2}**	**Au NP diameter, nm**
ProtoPasta	7.1(±0.9); *n* = 3	2.8×10^{8}	195(±97); *n* = 179
Amolen	9(±3); *n* = 3	8.9×10^{7}	264(±149); *n* = 206
BlackMagic 3D	5.2(±0.9); *n* = 3	1.3×10^{8}	222(±142); *n* = 260

S3. Cyclic voltammograms of 0.1 M KNO_3 for solvent window and geometric capacitance analysis

Figures S5 and S6 show representative capacitance and solvent window scans, respectively. All data was acquired in a three-electrode cell containing 0.1 M KNO_3 as the electrolyte.

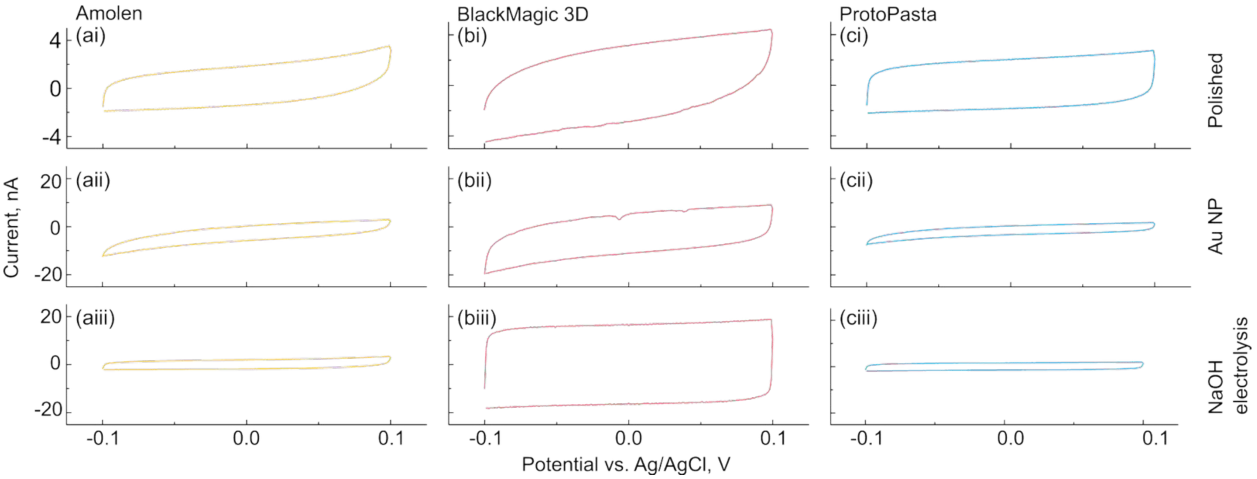

Figure S5. Representative capacitance CVs 0.1 M KNO_3 using alumina polished (a) Amolen, (b) BlackMagic 3D, and (c) ProtoPasta electrodes pretreated with (i) alumina polishing, (ii) Au NPs, and (iii) NaOH electrolysis. The experiments were performed in 0.1 M KNO_3 at a scan rate of 0.1 V s^{-1} in a three electrode cell containing a saturated Ag/AgCl reference and a Pt wire counter.

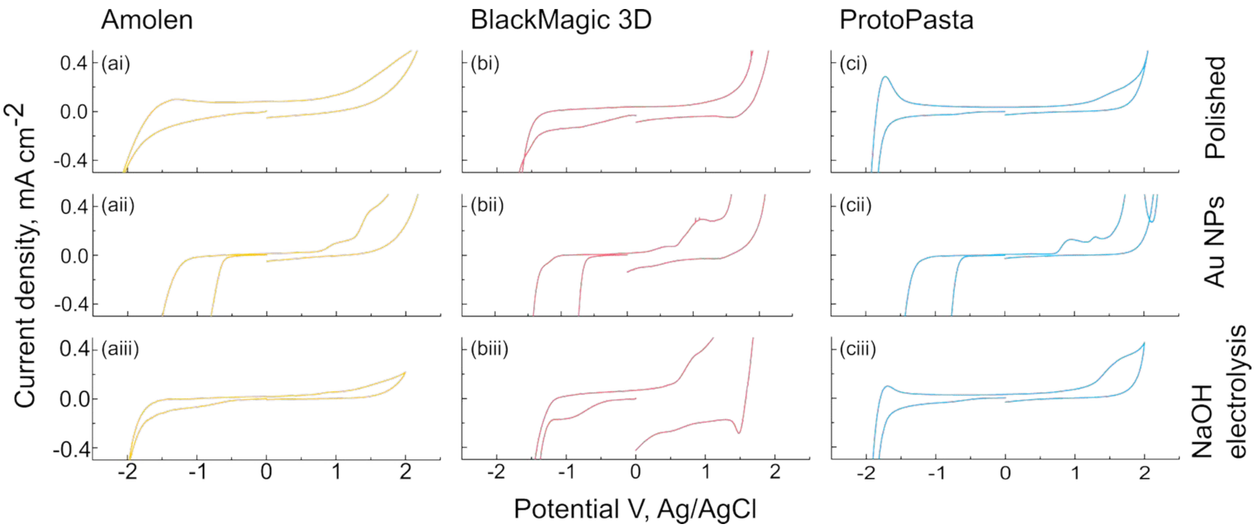

Figure S6. Representative solvent window CVs in 0.1 M KNO_3 using alumina polished (a) Amolen, (b) BlackMagic 3D, and (c) ProtoPasta electrodes pretreated with (i) alumina polishing, (ii) Au NPs, and (iii) NaOH electrolysis. The experiments were performed in 0.1 M KNO_3 at a scan rate of 0.1 V s^{-1} in a three electrode cell containing a saturated Ag/AgCl reference and a Pt wire counter.

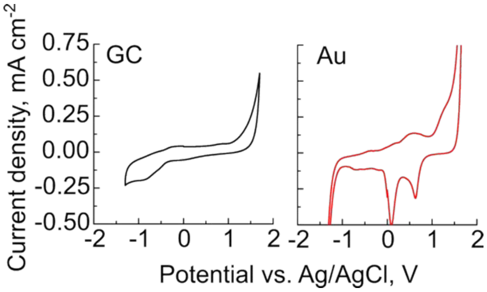

Figure S7. CVs in 0.1 M KNO_3 using GC (black trace) and Au (red trace) electrodes.

S4. Additional data for the analysis of $Ru(NH_3)_6^{3+}$ reduction on alumina polished electrodes.

Table S3. Comparison of slopes obtained from i_p versus $v^{1/2}$ plots for Amolen, BlackMagic 3D, and ProtoPasta electrodes.

**Filament**	**Theoretical slope,**[c] **A s$^{1/2}$ V$^{-1/2}$**	**Measured slope, A s$^{1/2}$ V$^{-1/2}$**		
		Polished	*Au NP*	*NaOH*
Amolen[a]	1.9•10^{-6}	8.9(±1.2)•10^{-7}; R^2 = 0.964	1.6(±0.7)•10^{-6}; R^2 = 0.994	n.d.[d]
BlackMagic 3D[b]	1.4•10^{-6}	1.49(±0.05)•10^{-6}; R^2 = 0.996	1.54(±0.02)•10^{-6}; R^2 = 0.999	2.41(±0.05)•10^{-6}; R^2 = 0.999
ProtoPasta[b]	1.6•10^{-6}	1.54(±0.04)•10^{-6}; R^2 = 0.998	1.70(±0.02)•10^{-6}; R^2 = 0.999	1.53(±0.02)•10^{-6}; R^2 = 0.999

[a] n = 4 independently prepared electrodes; [b] n = 3 independently prepared electrodes.
Values are reported as the mean ± one standard deviation.
[c] Theoretical slopes were calculated using Equations 2 (for BlackMagic 3D and ProtoPasta) or 3 (Amolen) from the main text using the following values; $n = 1$; $D = 8.8•10^{-6}$ cm^2 s^{-1}; $c_b = 1•10^{-6}$ mol cm^{-3} (1 mM); α = 0.5. Average electrode areas (A) from **Table S1** were used.
[d] Diffusive peaks were not detected.

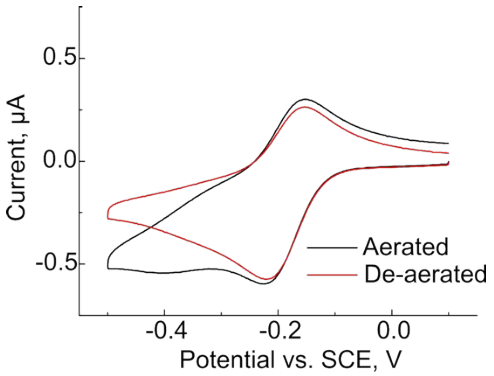

Figure S8. CVs of 1 mM $Ru(NH_3)_6^{3+}$ and 0.1 M KNO_3 using a BlackMagic 3D electrode pretreated with NaOH electrolysis in the presence (black trace) and absence of dissolved oxygen. Oxygen was removed by bubbling Ar through the solution for 30 minutes. v = 0.1 V s^{-1}; ref: SCE; counter: glassy carbon rod.

S5. Atomic force microscopy (AFM) images of BlackMagic 3D electrodes

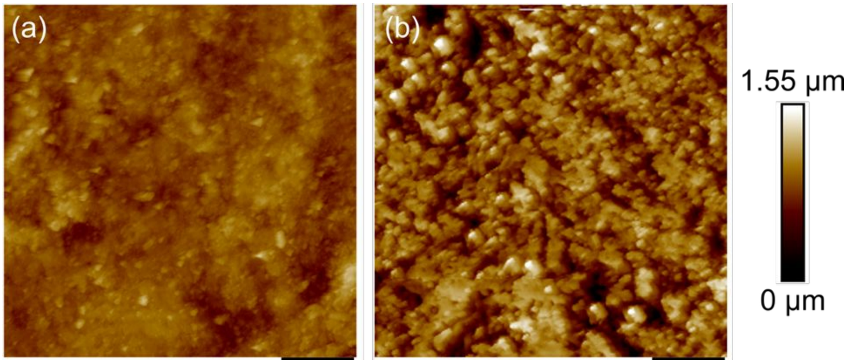

Figure S9. AFM images of (a) polished and (b) NaOH electrolyzed BlackMagic electrodes.

S6. Electrochemical impedance spectroscopy of polished electrodes.

To determine the uncompensated resistance (*R*u) of the electrodes, we performed electrochemical impedance spectroscopy (EIS) in an electrolyte containing 2.5 mM $K_3[Fe(CN)_6]$, 2.5 mM $K_4[Fe(CN)_6]$, and 0.1 M KNO_3 over a frequency range from 1-100,000 Hz with an amplitude of 10 mV and an applied potential of 0.3 V vs. SCE. The data was fit with a simple Randles circuit, which includes the uncompensated resistance in series with an RC capacitance element. Note, for the Amolen data, a constant phase element (CPE) was used in place of an the ideal capacitor in order to the fit to converge.

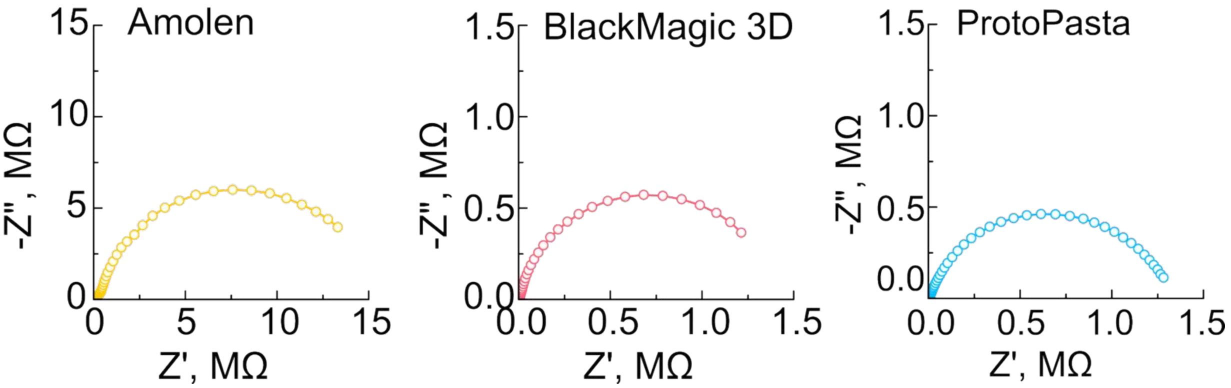

Figure S10. Representative Nyquist Plots for electrodes constructed from Amolen, BlackMagic 3D, and ProtoPasta

S7. Outer-sphere ET measurements using ferrocene methanol (FcMeOH)

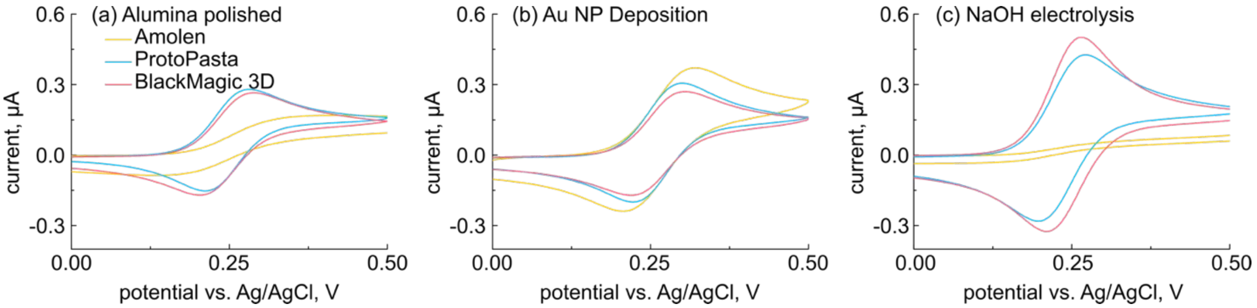

Figure S11. CVs for the oxidation of FcMeOH using Amolen (yellow), ProtoPasta (blue), and BlackMagic 3D (red) filament electrodes prepared by (a) alumina polishing, (b) Au NP electrodeposition, and (c) NaOH electrolysis.

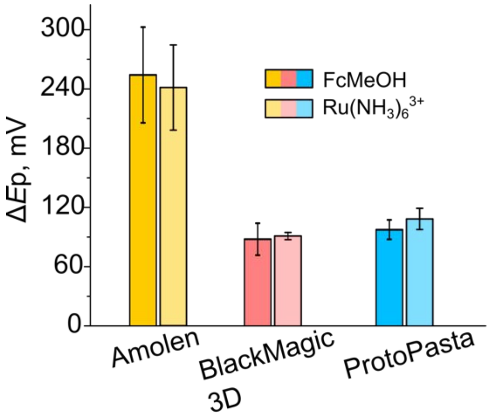

Figure S12. Statistical comparison of peak potential separations (ΔEp) measured with polished ProtoPasta, BlackMagic 3D, and Amolen electrodes for the oxidation of FcMeOH and reduction of $Ru(NH_3)_6^{3+}$.

S8. CV measurements performed with commercial glassy carbon and Au disk electrodes

We performed the voltammetric experiments for outer-sphere and inner-sphere redox species using commercial glassy carbon (GC) and Au disk electrodes. The experiments were performed under identical conditions to the main text except for the $Ru(NH_3)_6^{3+}$ reduction, which required de-oxygenating the solution with Ar for 15 minutes. The GC electrode was polished with alumina while the Au electrode was first polished with alumina then cycled in H_2SO_4 as described in the main text.

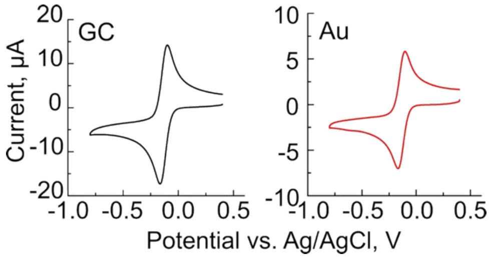

Figure S13. CVs showing the reduction of $Ru(NH_3)_6^{3+}$ on GC (black trace) and Au (red trace) electrodes.

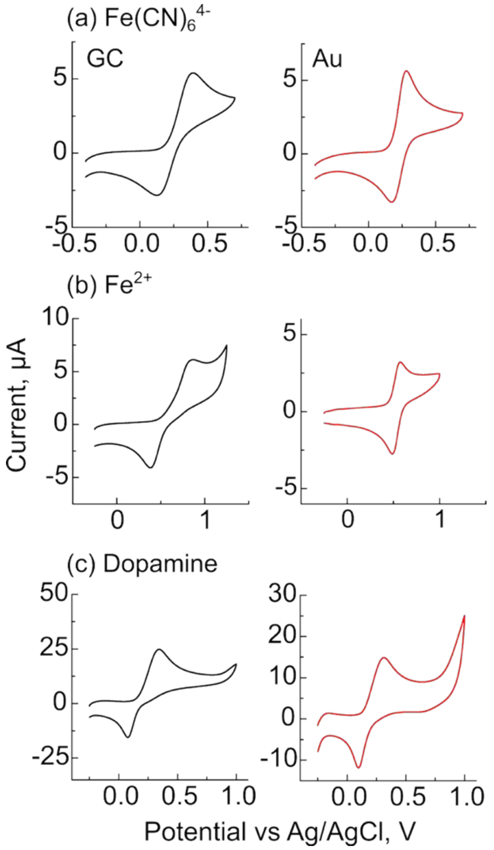

Figure S14. CVs showing the oxidation of (a) $Fe(CN)_6^{4-}$, (b) Fe^{2+}, and (c) dopamine on GC and Au electrodes.
